# Unequal causality between autoimmune thyroiditis and inflammatory bowel disease: a Mendelian randomization study

**DOI:** 10.3389/fendo.2024.1387482

**Published:** 2024-10-24

**Authors:** Siyang Bai, Yunfeng Yu, Xinyu Yang, Gang Hu, Jingyi Wu, Keke Tong, Yuman Yin, Juan Deng, Cong Chen, Chuanchuan Tan

**Affiliations:** ^1^ Department of Digestive Endoscopy, The First Hospital of Hunan University of Chinese Medicine, Changsha, Hunan, China; ^2^ School of Traditional Chinese Medicine, Hunan University of Chinese Medicine, Changsha, Hunan, China; ^3^ The Third School of Clinical Medicine, Zhejiang Chinese Medical University, Hangzhou, Zhejiang, China; ^4^ School of Basic Medicine, Guizhou University of Traditional Chinese Medicine, Guiyang, Guizhou, China

**Keywords:** autoimmune thyroiditis, inflammatory bowel disease, ulcerative colitis, Crohn’s disease, Mendelian randomization, FinnGen

## Abstract

**Objective:**

This study aims to analyze the causal relationship between autoimmune thyroiditis (AIT) and inflammatory bowel disease (IBD) using bidirectional Mendelian randomization (MR).

**Methods:**

Single nucleotide polymorphisms were obtained from FinnGen. Exposure-outcome causality was assessed using inverse variance weighted, MR-Egger, and weighted median. MR-Egger intercept, Cochran’s Q, and leave-one-out sensitivity analysis were used to evaluate horizontal pleiotropy, heterogeneity, and robustness, respectively.

**Results:**

Forward analysis revealed no significant association between AIT and the risk of ulcerative colitis (UC) (odds ratio [OR] 1.008, 95% confidence interval [CI] 0.986 to 1.03, *p =* 0.460) or Crohn’s disease (CD) (OR 0.972, 95% CI 0.935 to 1.010, *p* = 0.143). Reverse analysis showed that UC (OR 0.961, 95% CI 0.783 to 1.180, *p* = 0.707) was not associated with AIT risk, while CD (OR 2.371, 95% CI 1.526 to 3.683, *p* < 0.001) was linked to an increased risk of AIT. Intercept analysis and Cochran’s Q test indicated no horizontal pleiotropy or heterogeneity. Sensitivity analysis confirmed the robustness of the MR results.

**Conclusion:**

This MR analysis suggests that CD, but not UC, is a risk factor for AIT, whereas AIT is not associated with the risk of IBD. Proactive prevention and treatment of CD can help mitigate the risk of AIT.

## 1 Introduction

Inflammatory bowel disease (IBD) is an immune-mediated chronic inflammatory disease of the gastrointestinal tract, primarily consisting of Crohn’s disease (CD) and ulcerative colitis (UC) (1, 2). Epidemiological studies show that the prevalence of IBD is more than 0.3% in Western countries and continues to rise annually (3, 4). IBD presents with gastrointestinal symptoms, including abdominal pain, diarrhea, and rectal bleeding, which may be accompanied by systemic symptoms or extraintestinal organ involvement in some patients (5, 6). Chronic and recurrent inflammation in IBD elevates the risk of cardiovascular disease such as myocardial infarction, heart failure, stroke, and mental disorders (7, 8), imposing significant burdens on individuals, families, and society.

Autoimmune thyroiditis (AIT) is an autoimmune disease characterized by abnormal thyroid function resulting from the immune system’s attack on thyroid tissue (9, 10). It is characterized by overproduction of thyroid autoantibodies and lymphocytic infiltration in thyroid tissue (11). Epidemiological research shows that the prevalence of AIT is 3% to 5% and has shown a consistent increase over the years (12). Fatigue, bradycardia, and chills are the main clinical manifestations of AIT and contribute significantly to a decline in patients’ quality of life (13). As research progressed, investigators identified potential links between AIT and IBD (14), including common genetic factors, immune abnormalities, and inflammatory processes (14–16). In past observational studies, several studies have explored the relationship between IBD and AIT (17–20), but the causal relationship is unclear due to limitations in study design. Therefore, more robust methods are warranted to investigate this association thoroughly.

Mendelian randomization (MR) is an innovative method for analyzing the causal effect of exposures on outcomes by leveraging genetic variation (21). Compared to traditional methods, MR is less susceptible to issues like reverse causation and confounding biases (22). Therefore, in this study, we employed MR to assess the causal relationship between AIT and IBD and conducted an analysis integrating clinical data.

## 2 Materials and methods

### 2.1 Study design

**Figure 1:**
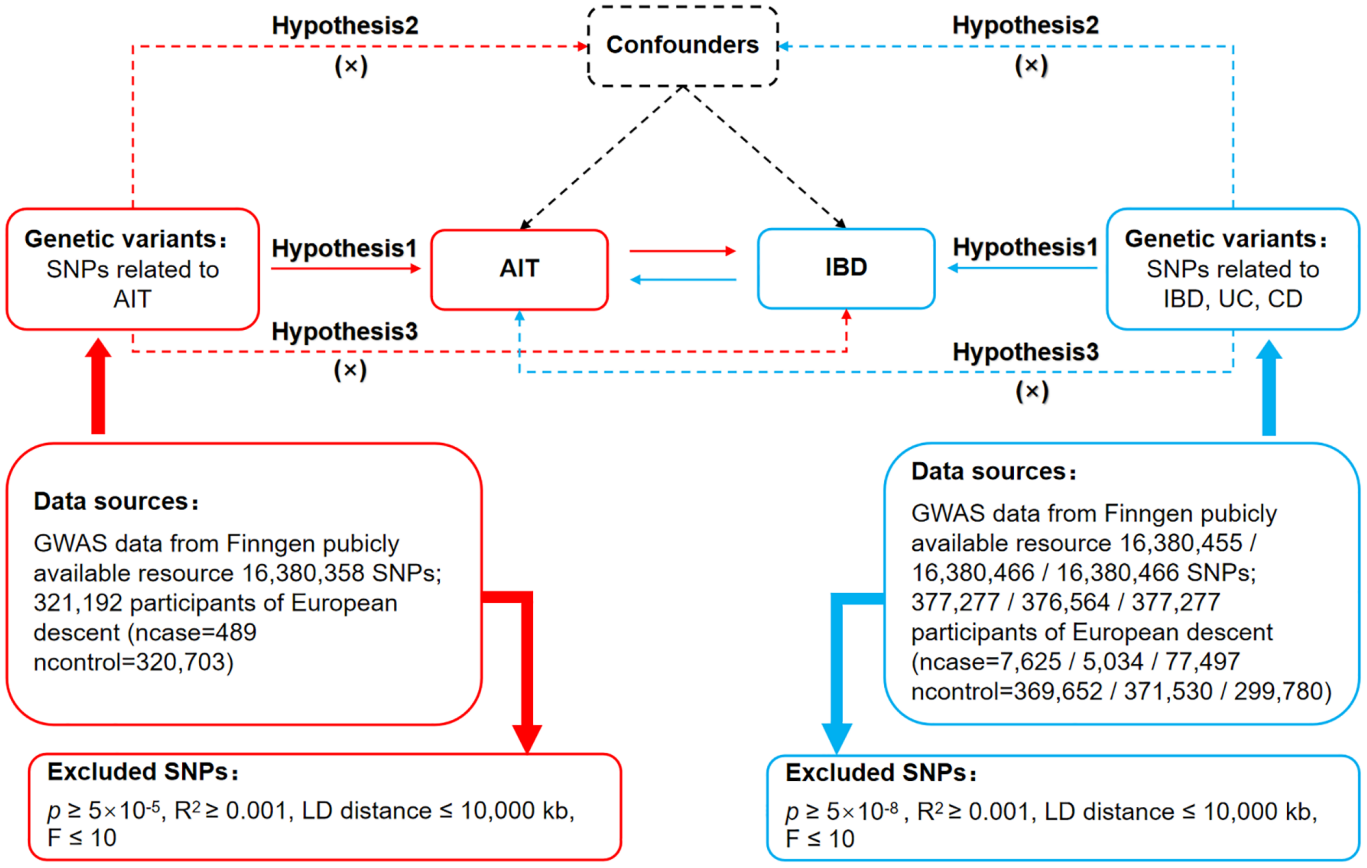
Bidirectional MR design for causal analysis of AIT and IBD. AIT, autoimmune thyroiditis; IBD, inflammatory bowel disease.

The MR study is grounded in three fundamental assumptions (23), as illustrated in **Figure 1**. The association assumption necessitates that single nucleotide polymorphisms (SNPs) are strongly correlated with exposure. The independence assumption requires that SNPs are independent of confounding variables. The exclusivity assumption demands that the SNPs influence the outcome solely through the exposure and not via other pathways.

### 2.2 Data sources

FinnGen (www.finngen.fi/fi) is a publicly available database, which compiles samples from the nationwide network of Finnish bio-banks and digital healthcare data from the national health registry (24). FinnGen provided datasets for AIT, IBD, UC, and CD in this MR study. Among them, the AIT dataset was finngen-R9-E4-THYROIDITAUTOIM, including 321,192 Europeans; the IBD dataset was finngen-R9-K11-IBD-STRICT, encompassing 377,277 Europeans; the UC dataset was finngen-R9-K11-UC-STRICT2, comprising 376,564 Europeans; and the CD dataset was finngen-R9-RX-CROHN-1STLINE, containing 377,277 Europeans, as detailed in **Table 1**. As these datasets are publicly available, this MR study does not require additional ethical approval.

**Table 1:** Details of the datasets included in the Mendelian randomization.

Year	Trait	GWAS ID	Population	Sample size	Web source
2023	AIT	finngen-R9-E4-THYROIDITAUTOIM	European	321,192	www.finngen.fi/fi
2021	IBD	finngen-R9-K11-IBD-STRICT	European	377,277	www.finngen.fi/fi
2021	UC	finngen-R9-K11-UC-STRICT2	European	376,564	www.finngen.fi/fi
2021	CD	finngen-R9-RX-CROHN-1STLINE	European	377,277	www.finngen.fi/fi

AIT, autoimmune thyroiditis; IBD, inflammatory bowel disease; UC, ulcerative colitis; CD, Crohn’s disease.

### 2.3 Selection of genetic instrument variables

First, a significance level of *p* < 5 × 10^-5^ was applied to search for SNPs in the AIT dataset, and *p* < 5 ×10^-8^ for SNPs in IBD and its subtypes, adhering to the association assumption. Second, constraints of *kb* = 10,000 and *R^2^* < 0.001 were imposed to mitigate the interference of linkage disequilibrium. Third, a limit of *F* ≤ 10 was set to identify SNPs with strong correlation, where *F* = [*R* ^2^/(1 – *R* ^2^)]*[(*N* – *K* – 1)/*k*], with *K* representing the number of paired samples, *N* the total number of samples, and *R^2^* the cumulative explained variance. Fourth, PhenoScanner (www.phenoscanner.medschl.cam.ac.uk) and Google Scholar were used to exclude SNPs with confounding factors to fulfill the independence assumption. Fifth, mismatched SNPs were excluded based on the effect allele frequency during the adjustment of allele directions between exposure and outcome. Sixth, MR-Pleiotropy Residual Sum and Outlier method (MR-PRESSO) was employed to exclude outlier SNPs (*p* < 1) to ensure the accuracy of causal inference.

### 2.4 Data analysis

The STROBE-MR guidelines served as a guiding method (25). R 4.3.1 software with the “TwoSampleMR (0.5.7)” package installed, was used for all operations of MR analysis. Inverse variance weighted (IVW) was chosen as the primary tool due to its ability to allow unbiased causal analysis without pleiotropy (26). Weighted median, sensitive to outliers, and MR-Egger, capable of analyzing data in the presence of pleiotropy, were set as secondary tools. The intercept of MR-Egger was also utilized to analyze horizontal pleiotropy, necessary to satisfy the exclusivity assumption (*p* ≥ 0.05). Cochran’s Q and leave-one-out analyses were employed for heterogeneity and sensitivity analysis, respectively. There was no heterogeneity in the results at *p* ≥ 0.05, and the results were robust when the combined effect sizes were not significantly altered.

## 3 Results

### 3.1 Genetic instrument variables

Following association, independence, and exclusivity tests, 53 SNPs for AIT, 31 SNPs for IBD, 25 SNPs for UC, and 28 SNPs for CD were included in this study, as shown in **Supplementary Table S1**. After removing mismatched and outlier SNPs, the SNPs for each exposure-outcome group are presented in **Supplementary Table S2**.

### 3.2 Bidirectional MR analysis

#### 3.2.1 Impact of AIT on IBD

**Figure 2:**
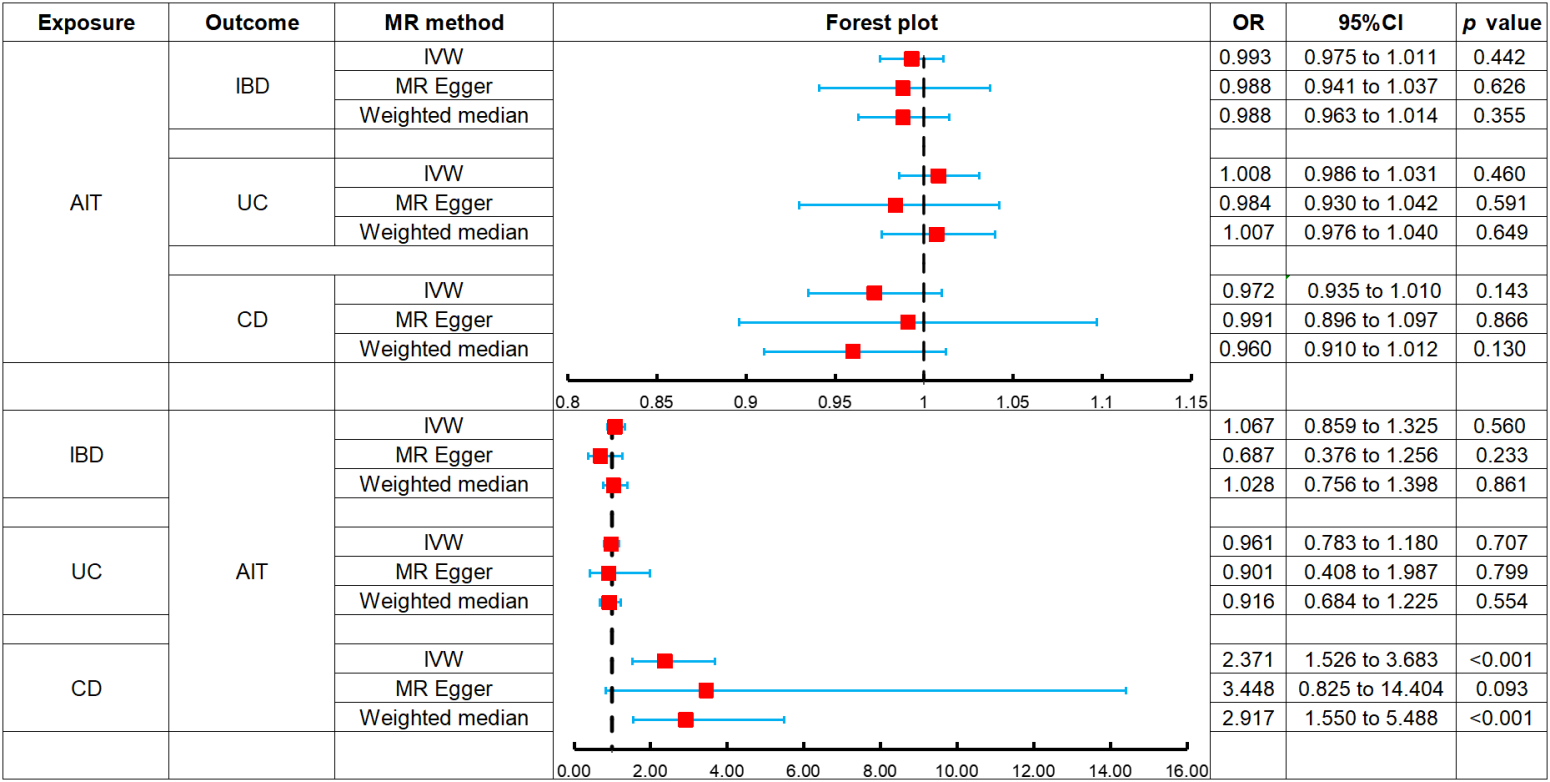
Forest plots of MR analysis on the causal relationship between AIT and IBD. AIT, autoimmune thyroiditis; IBD, inflammatory bowel disease; UC, ulcerative colitis; CD, Crohn’s disease.

**Figure 3:**
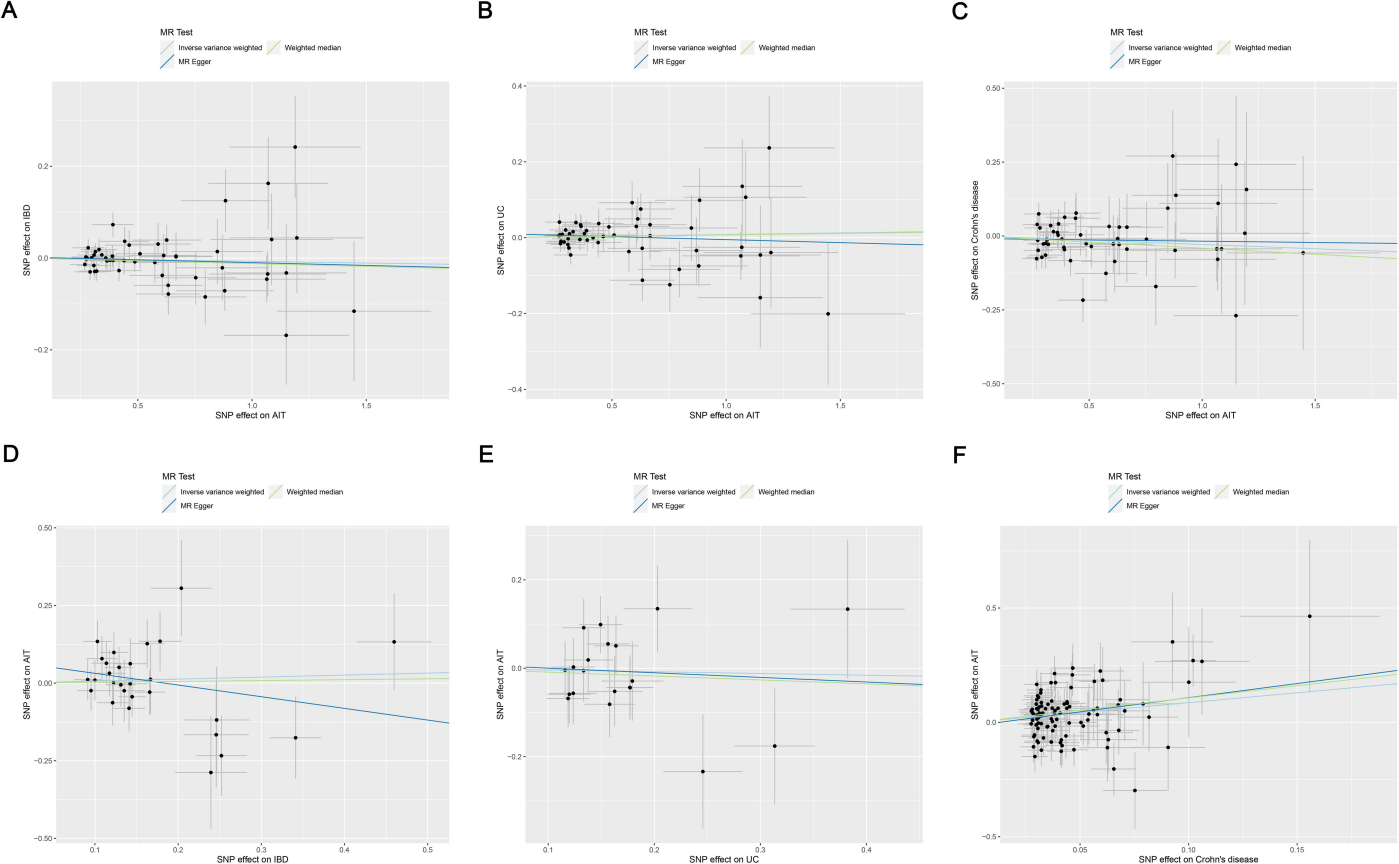
Scatter plots of MR analysis on the causal relationship between AIT and IBD. **(A)** AIT on IBD; **(B)** AIT on UC; **(C)** AIT on CD; **(D)** IBD on AIT; **(E)** UC on AIT; **(F)** CD on AIT. CD on AIT. AIT, autoimmune thyroiditis; IBD, inflammatory bowel disease; UC, ulcerative colitis; CD, Crohn’s disease.

The MR analysis indicated no association between AIT and the risk of IBD: IVW (OR, 0.993; 95% CI, 0.975 to 1.011; *p* = 0.442), MR-Egger (OR, 0.988; 95% CI, 0.941 to 1.037; *p* = 0.626), and weighted median (OR, 0.988; 95% CI, 0.963 to 1.014; *p* = 0.355), as the forest plot depicted in **Figure 2** and the scatter plot depicted in **Figure 3A**. Moreover, no horizontal pleiotropy was observed in the result (*p* = 0.833), as detailed in **Supplementary Table S3**.

#### 3.2.2 Impact of AIT on UC

The MR analysis revealed no association between AIT and the risk of UC: IVW (OR, 1.008; 95% CI, 0.986 to 1.031; *p* = 0.460), MR-Egger (OR, 0.984; 95% CI, 0.930 to 1.042; *p* = 0.591), and weighted median (OR, 1.007; 95% CI, 0.976 to 1.040; *p* = 0.649), as the forest plot depicted in **Figure 2** and the scatter plot depicted in **Figure 3B**. Moreover, no horizontal pleiotropy was observed in the result (*p* = 0.375), as detailed in **Supplementary Table S3**.

#### 3.2.3 Impact of AIT on CD

The MR analysis demonstrated no association between AIT and the risk of CD: IVW (OR, 0.972; 95% CI, 0.935 to 1.010; *p* = 0.143), MR-Egger (OR, 0.991; 95% CI, 0.896 to 1.097; *p* = 0.866), and weighted median (OR, 0.960; 95% CI, 0.910 to 1.012; *p* = 0.130), as the forest plot depicted in **Figure 2** and the scatter plot depicted in **Figure 3**. Moreover, no horizontal pleiotropy was observed in the results (*p* = 0.676), as detailed in **Supplementary Table S3**.

#### 3.2.4 Impact of IBD on AIT

The MR analysis revealed no association between IBD and the risk of AIT: IVW (OR, 1.067; 95% CI, 0.859 to 1.325; *p* = 0.560), MR-Egger (OR, 0.687; 95% CI, 0.376 to 1.256; *p* = 0.233), and weighted median (OR, 1.028; 95% CI, 0.756 to 1.398; *p* = 0.861), as the forest plot depicted in **Figure 2** and the scatter plot depicted in **Figure 3**. Moreover, no horizontal pleiotropy was observed in the results (*p* = 0.139), as detailed in **Supplementary Table S3**.

#### 3.2.5 Impact of UC on AIT

The MR analysis demonstrated no association between UC and the risk of AIT: IVW (OR, 0.961; 95% CI, 0.783 to 1.180; *p* = 0.707), MR-Egger (OR, 0.901; 95% CI, 0.408 to 1.987; *p* = 0.799), and weighted median (OR, 0.916; 95% CI, 0.684 to 1.225; *p* = 0.554), as the forest plot depicted in **Figure 2** and the scatter plot depicted in **Figure 3**. Moreover, no horizontal pleiotropy was observed in the results (*p* = 0.869), as detailed in **Supplementary Table S3**.

#### 3.2.6 Impact of CD on AIT

Both IVW (OR, 2.371; 95% CI, 1.526 to 3.683; *p* < 0.001) and weighted median (OR, 2.917; 95% CI, 1.550 to 5.488; *p* < 0.001) indicated that CD was associated with an increased risk of AIT, while MR-Egger (OR, 3.448; 95% CI, 0.825 to 14.404; *p* = 0.093) did not observe such a causal relationship, as the forest plot depicted in **Figure 2** and the scatter plot depicted in **Figure 3F**. Moreover, no horizontal pleiotropy was observed in the results (*p* = 0.560), as detailed in **Supplementary Table S3**.

### 3.3 Heterogeneity and sensitivity analysis

**Figure 4:**
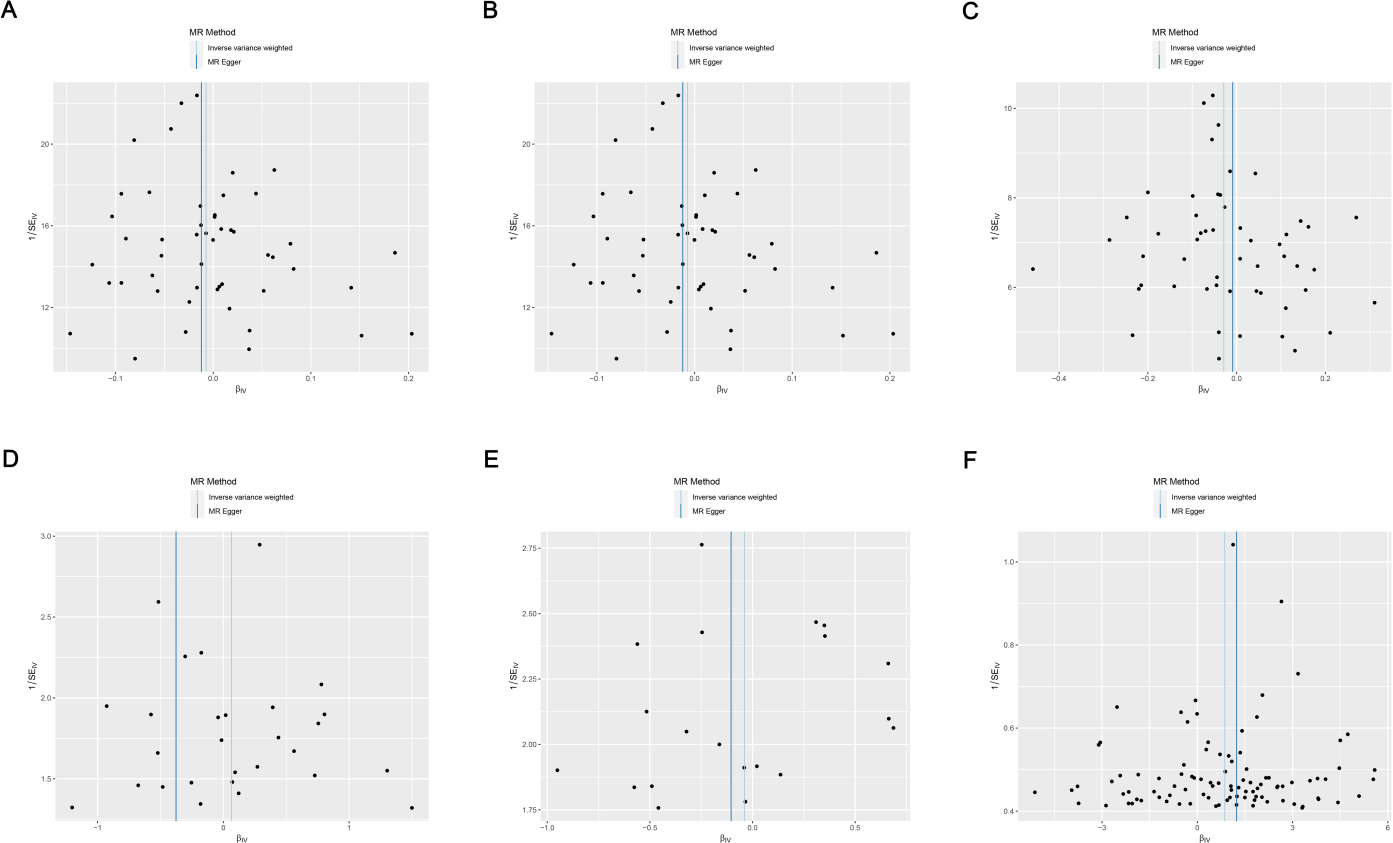
Funnel plots of heterogeneity analysis on the causal relationship between AIT and IBD. **(A)** AIT on IBD; **(B)** AIT on UC; **(C)** AIT on CD; **(D)** IBD on AIT; **(E)** UC on AIT; **(F)** CD on AIT. AIT, autoimmune thyroiditis; IBD, inflammatory bowel disease; UC, ulcerative colitis; CD, Crohn’s disease.

**Figure 5:**
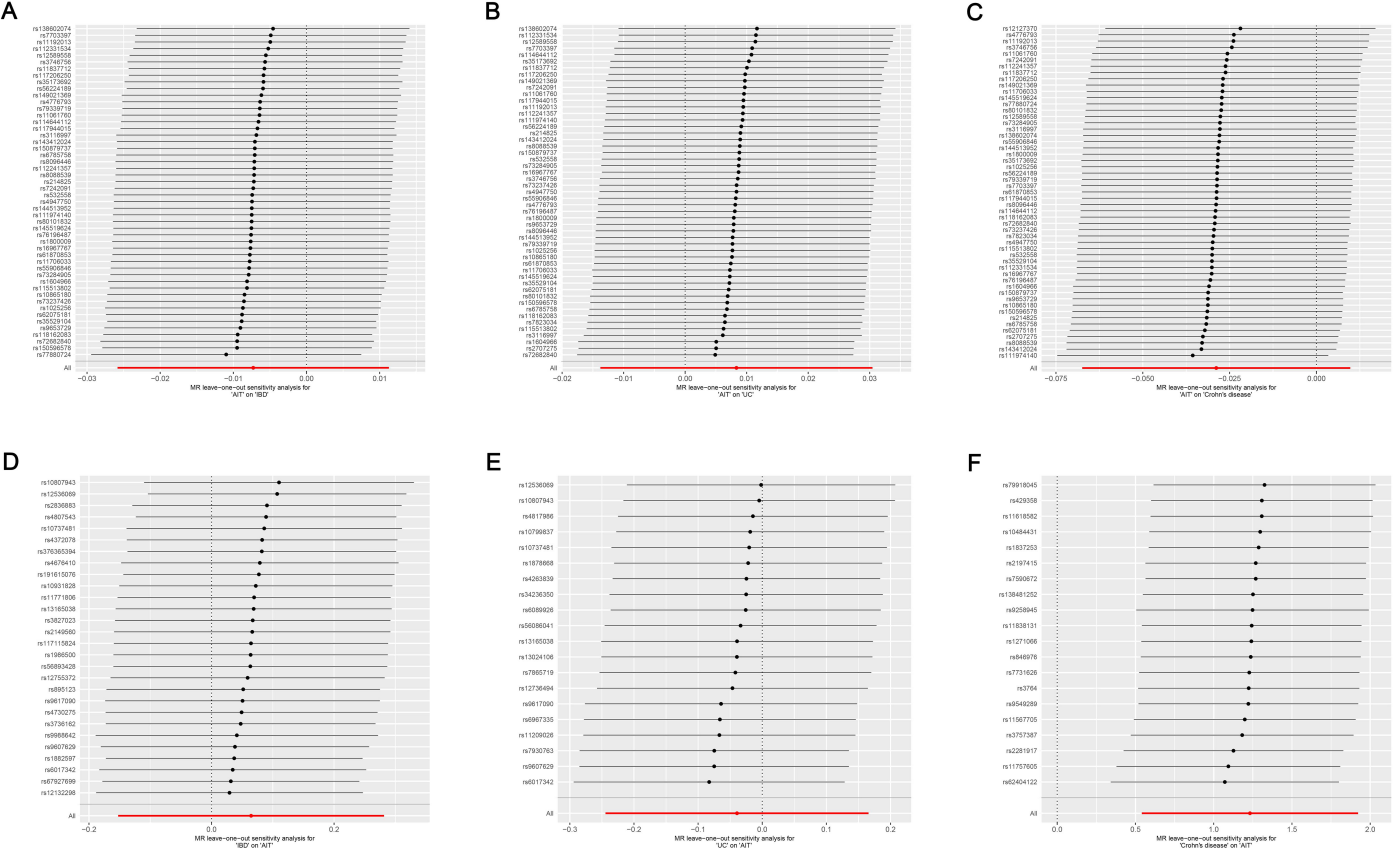
Leave-one-out sensitivity analysis on the causal relationship between AIT and IBD. **(A)** AIT on IBD; **(B)** AIT on UC; **(C)** AIT on CD; **(D)** IBD on AIT; **(E)** UC on AIT; **(F)** CD on AIT. AIT, autoimmune thyroiditis; IBD, inflammatory bowel disease; UC, ulcerative colitis; CD, Crohn’s disease.

Cochran’s Q test suggested no heterogeneity in the MR analysis results (*p* ≥ 0.05), as the funnel plots depicted in **Figure 4** and the results of Cochran’s Q depicted in **Supplementary Table S4**. Sensitivity analysis confirmed the robustness of the results, as shown in **Figure 5**.

## 4 Discussion

As an autoimmune disease, AIT is frequently associated with other organ-specific or systemic autoimmune diseases (27, 28). Recently, the correlation between AIT and IBD has received increasing attention from researchers (16, 18). Relevant studies indicate that patients with AIT exhibit a significantly elevated risk for autoimmune diseases such as autoimmune gastritis, rheumatoid arthritis, and celiac disease but not an increased risk of IBD (19). However, some studies suggest that thyroid dysfunction often affects the gastrointestinal system, potentially exacerbating IBD conditions (20). The association between AIT and IBD remains contentious. This MR analysis indicated that CD, but not UC, was associated with an increased risk of AIT, while AIT was not associated with either UC or CD risk. These results were free of horizontal pleiotropy and heterogeneity, and were confirmed to be robust by sensitivity analysis.

Our results demonstrate that AIT is not associated with the risk of IBD and its subtypes. Although reports on to the effect of AIT on the risk of IBD are scarce, the available clinical study supports our findings. The cohort study by Fallahi et al. (19) assessed the prevalence of other autoimmune diseases in 3,069 white AIT patients between 1993 and 2008. And they also found that the prevalence of UC and CD in AIT patients was 0.55% and 0.39%, respectively, which was not significantly different from that of the general population controls (19). This evidence points to the fact that AIT is not a risk factor for IBD. However, due to the limited clinical evidence available, further studies are necessary to validate this result.

Additionally, previous clinical studies have mainly concluded that IBD and UC are not associated with the risk of AIT, which supports our findings. A cross-sectional study in California showed that although IBD increased the risk of autoimmune diseases such as rheumatoid arthritis, multiple sclerosis, and psoriasis, it did not elevate the prevalence of AIT compared to controls (0.25% *vs.* 0.21%) (29). An Israeli retrospective study analyzed the prevalence of autoimmune diseases in 12,625 patients with IBD and found that IBD was associated with all autoimmune diseases except AIT (30). Shizuma et al. (31) reported no significant difference in the prevalence of thyroid dysfunction between patients with IBD and the general population, which indirectly supports the idea that IBD is not associated with the risk of AIT. Moreover, Bernstein et al. (32) further noted that the risk of AIT was comparable to that of controls in both UC patients (PR, 1.58; 95% CI, 0.79 to 3.20) and CD patients (PR, 0.96; 95% CI, 0.47 to 2.00) after analyzing 8,072 IBD cases in the University of Manitoba IBD Database. Casella et al. (33) also found a relatively low incidence of hypothyroidism in patients with UC (1.9%), suggesting that UC is not associated with AIT risk. In summary, these pieces of evidence point to the fact that IBD is not a risk factor for AIT, which is consistent with the results of this MR analysis.

Interestingly, our study revealed an association between CD and an increased risk of AIT, supported by several clinical research. Shah et al. (16) first reported three cases of AIT associated with CD in 1998, suggesting that CD may be involved in the pathogenesis of AIT. Messina et al. (34) noted that patients with CD were more likely to have increased thyroid volume and uneven parenchymal structure compared to normal individuals, and these abnormal changes did not completely normalize even after treatment. A cross-sectional study by Bardella et al. (18) reported that the incidence of AIT in patients with UC was comparable to that of controls (OR, 2.2; 95% CI, 0.3 to 7.8), whereas the incidence of AIT in patients with CD was significantly higher than that of controls (OR, 4.4; 95% CI, 1.2 to 11.0). Moreover, the logistic regression analysis by Kappelman et al. (35) found that the risk of hypothyroidism was significantly higher in patients with CD than in the general population (OR 2.9, 95% CI 1.4 to 6.1), while the risk of hypothyroidism in UC patients was similar to that in the general population. These pieces of evidence point to CD, rather than UC, as a potential risk factor for AIT, consistent with our analysis results. Meanwhile, it also suggests that the insignificant effect of IBD on AIT is mediated by UC.

The differential impact of UC and CD on the risk of AIT may stem from differences in their pathogenesis (36). The pathology of UC is characterized by continuous and diffuse inflammation of the colonic mucosa (37). It is marked by an atypical T helper cell (Th2)-like response, secretion of cytokines such as IL-13, and increased expression of Th17 (36, 38). However, the pathological manifestations of CD include transmural inflammation and epithelial granulomas (39). It is characterized by a helper T-cell (Th1) response, elevated levels of IFN-γ and TNF-α, and tissue infiltration by Th17 cells (40). AIT shares an immune response with CD, and their pathogenesis involves CD4^+^ T lymphocytes of the Th1 phenotype, contrasting with the Th2 phenotype of UC (18, 40, 41). IFN-γ secreted by Th1 makes the intestinal barrier more susceptible to damage by disrupting tight junction proteins (42), subsequently affecting other organs in the body, and this may be a potential mechanism by which CD increases the risk of AIT.

To further explore the genetic mechanisms through which CD influences AIT, we investigated 20 SNPs related to CD-AIT. Among them, only one SNP has been reported in the literature. The rs4657041 was the lead intronic cis-pQTL for FCGR2A and FCGR2B, which was shared with UC, systemic lupus erythematosus and various cell surface markers of different immune cell populations (43). However, no studies have reported an association between rs4657041 and either CD or AIT. More research is needed in the future to explore the roles and significance of these SNPs.

While this MR analysis enhances the genetic evidence, there are inevitably some limitations. First, as the data were derived from Europeans, our findings may not extrapolate to elucidate the effects of AIT and IBD in other ancestry diversity. Second, this study identified CD as a risk factor for AIT, but the mechanism remains unclear. Third, there is an increased potential for selectivity bias, as there may be under-recognized confounding variables. Acknowledging these limitations, we encourage future researchers to enhance the GWAS database with racially diverse MR research to promote health equity. Moreover, higher-quality future studies are needed to elucidate the causal effects of AIT and IBD, and to explore the molecular biological mechanisms through which CD elevates the risk of AIT.

## 5 Conclusion

This MR analysis suggests that CD, but not UC, is a risk factor for AIT, whereas AIT is not associated with IBD risk. Active prevention and treatment of CD can help reduce the risk of AIT. More studies are needed to explore the intrinsic link and mechanism of action between AIT and IBD in the future.

## Data Availability

The original contributions presented in the study are included in the article/**Supplementary Material**. Further inquiries can be directed to the corresponding authors.
