# Supplementary material for: Unequal causality between autoimmune thyroiditis and inflammatory bowel disease: a Mendelian randomization study
Source: Front Endocrinol (Lausanne). 2024 Oct 24;15:1387482. doi: 10.3389/fendo.2024.1387482 (PMC11540656; doi:10.3389/fendo.2024.1387482)
Supplement: Supplementary file 1 [file Table1.docx]

**Supplementary Table S1.1** Characteristics of instrumental variables for AIT.

|  | **SNP** | **EA** | **OA** | **Samplesize** | **SE** | **β** | **id.exposure** | **EAF** | ***p* value** | **R^2^** | **F - statistic** |
| --- | --- | --- | --- | --- | --- | --- | --- | --- | --- | --- | --- |
| 1 | rs145519624 | C | A | 321192 | 0.1464 | 0.613155 | finngen-R9-E4-THYROIDITAUTOIM | 0.033059 | 2.81E-05 | 0.024036 | 7910.312 |
| 2 | rs115513802 | C | T | 321192 | 0.144354 | 0.588563 | finngen-R9-E4-THYROIDITAUTOIM | 0.032844 | 4.56E-05 | 0.022008 | 7227.679 |
| 3 | rs72682840 | T | C | 321192 | 0.151106 | -0.62632 | finngen-R9-E4-THYROIDITAUTOIM | 0.064804 | 3.40E-05 | 0.047547 | 16033.87 |
| 4 | rs138602074 | T | C | 321192 | 0.212512 | -0.88033 | finngen-R9-E4-THYROIDITAUTOIM | 0.037213 | 3.44E-05 | 0.055531 | 18884.84 |
| 5 | rs12127370 | G | A | 321192 | 0.108538 | 0.472716 | finngen-R9-E4-THYROIDITAUTOIM | 0.070257 | 1.33E-05 | 0.029193 | 9658.507 |
| 6 | rs2707275 | T | C | 321192 | 0.062625 | 0.270325 | finngen-R9-E4-THYROIDITAUTOIM | 0.447166 | 1.58E-05 | 0.03613 | 12039.53 |
| 7 | rs10865180 | A | G | 321192 | 0.079889 | 0.329956 | finngen-R9-E4-THYROIDITAUTOIM | 0.157956 | 3.62E-05 | 0.028961 | 9579.4 |
| 8 | rs35529104 | T | C | 321192 | 0.261141 | 1.07077 | finngen-R9-E4-THYROIDITAUTOIM | 0.006823 | 4.13E-05 | 0.015539 | 5069.804 |
| 9 | rs114644112 | G | A | 321192 | 0.177922 | 0.753603 | finngen-R9-E4-THYROIDITAUTOIM | 0.023708 | 2.28E-05 | 0.02629 | 8672.096 |
| 10 | rs11706033 | T | C | 321192 | 0.091815 | -0.38873 | finngen-R9-E4-THYROIDITAUTOIM | 0.163309 | 2.30E-05 | 0.041295 | 13834.83 |
| 11 | rs6785758 | A | G | 321192 | 0.081055 | 0.364884 | finngen-R9-E4-THYROIDITAUTOIM | 0.144337 | 6.74E-06 | 0.032887 | 10922.06 |
| 12 | rs80101832 | G | A | 321192 | 0.071367 | -0.29745 | finngen-R9-E4-THYROIDITAUTOIM | 0.300906 | 3.07E-05 | 0.037224 | 12418.1 |
| 13 | rs77880724 | T | A | 321192 | 0.092581 | 0.39086 | finngen-R9-E4-THYROIDITAUTOIM | 0.104424 | 2.42E-05 | 0.028574 | 9447.734 |
| 14 | rs79339719 | A | C | 321192 | 0.144127 | 0.606856 | finngen-R9-E4-THYROIDITAUTOIM | 0.03477 | 2.55E-05 | 0.024719 | 8140.77 |
| 15 | rs7703397 | C | A | 321192 | 0.070007 | 0.311465 | finngen-R9-E4-THYROIDITAUTOIM | 0.685414 | 8.62E-06 | 0.041835 | 14023.7 |
| 16 | rs112241357 | A | G | 321192 | 0.138244 | 0.574445 | finngen-R9-E4-THYROIDITAUTOIM | 0.035717 | 3.25E-05 | 0.022731 | 7470.657 |
| 17 | rs1604966 | C | G | 321192 | 0.066388 | 0.342512 | finngen-R9-E4-THYROIDITAUTOIM | 0.274548 | 2.48E-07 | 0.046731 | 15745.46 |
| 18 | rs76196487 | G | A | 321192 | 0.200174 | 0.848037 | finngen-R9-E4-THYROIDITAUTOIM | 0.01371 | 2.27E-05 | 0.019449 | 6370.608 |
| 19 | rs3116997 | A | G | 321192 | 0.090937 | -0.48694 | finngen-R9-E4-THYROIDITAUTOIM | 0.17336 | 8.57E-08 | 0.067959 | 23419.26 |
| 20 | rs4947750 | C | T | 321192 | 0.160791 | 0.667293 | finngen-R9-E4-THYROIDITAUTOIM | 0.025909 | 3.32E-05 | 0.022475 | 7384.84 |
| 21 | rs55906846 | G | C | 321192 | 0.105867 | 0.509594 | finngen-R9-E4-THYROIDITAUTOIM | 0.070468 | 1.48E-06 | 0.03402 | 11311.79 |
| 22 | rs118162083 | G | A | 321192 | 0.287166 | 1.18895 | finngen-R9-E4-THYROIDITAUTOIM | 0.005652 | 3.47E-05 | 0.01589 | 5186.235 |
| 23 | rs7823034 | A | G | 321192 | 0.079897 | -0.36298 | finngen-R9-E4-THYROIDITAUTOIM | 0.843796 | 5.54E-06 | 0.034731 | 11556.57 |
| 24 | rs56224189 | T | G | 321192 | 0.150782 | 0.632792 | finngen-R9-E4-THYROIDITAUTOIM | 0.031087 | 2.71E-05 | 0.024122 | 7939.399 |
| 25 | rs61870853 | C | A | 321192 | 0.267453 | 1.08581 | finngen-R9-E4-THYROIDITAUTOIM | 0.00694 | 4.91E-05 | 0.01625 | 5305.715 |
| 26 | rs11192013 | C | T | 321192 | 0.069748 | -0.291 | finngen-R9-E4-THYROIDITAUTOIM | 0.313031 | 3.02E-05 | 0.036421 | 12140.06 |
| 27 | rs117206250 | T | A | 321192 | 0.274268 | 1.14957 | finngen-R9-E4-THYROIDITAUTOIM | 0.006069 | 2.77E-05 | 0.015944 | 5204.031 |
| 28 | rs1800009 | C | T | 321192 | 0.063752 | 0.272537 | finngen-R9-E4-THYROIDITAUTOIM | 0.361876 | 1.91E-05 | 0.034304 | 11409.52 |
| 29 | rs35173692 | A | T | 321192 | 0.257188 | -1.06458 | finngen-R9-E4-THYROIDITAUTOIM | 0.028921 | 3.48E-05 | 0.063658 | 21836.3 |
| 30 | rs150879737 | T | C | 321192 | 0.264898 | 1.15009 | finngen-R9-E4-THYROIDITAUTOIM | 0.0065 | 1.41E-05 | 0.017083 | 5582.243 |
| 31 | rs11061760 | C | T | 321192 | 0.075124 | 0.30745 | finngen-R9-E4-THYROIDITAUTOIM | 0.192727 | 4.27E-05 | 0.029413 | 9733.513 |
| 32 | rs11837712 | C | G | 321192 | 0.179719 | 0.795142 | finngen-R9-E4-THYROIDITAUTOIM | 0.0191 | 9.67E-06 | 0.023691 | 7793.932 |
| 33 | rs1025256 | G | A | 321192 | 0.065123 | 0.314269 | finngen-R9-E4-THYROIDITAUTOIM | 0.308637 | 1.39E-06 | 0.042149 | 14133.55 |
| 34 | rs73237426 | T | A | 321192 | 0.106278 | 0.462271 | finngen-R9-E4-THYROIDITAUTOIM | 0.074065 | 1.36E-05 | 0.02931 | 9698.311 |
| 35 | rs16967767 | T | C | 321192 | 0.29416 | 1.19627 | finngen-R9-E4-THYROIDITAUTOIM | 0.00482 | 4.77E-05 | 0.013728 | 4470.766 |
| 36 | rs12589558 | C | T | 321192 | 0.074585 | 0.319573 | finngen-R9-E4-THYROIDITAUTOIM | 0.194767 | 1.83E-05 | 0.032034 | 10629.4 |
| 37 | rs117944015 | T | C | 321192 | 0.337467 | 1.44667 | finngen-R9-E4-THYROIDITAUTOIM | 0.002934 | 1.81E-05 | 0.012244 | 3981.455 |
| 38 | rs144513952 | A | G | 321192 | 0.158488 | 0.667098 | finngen-R9-E4-THYROIDITAUTOIM | 0.026679 | 2.56E-05 | 0.023112 | 7598.867 |
| 39 | rs149021369 | T | C | 321192 | 0.256528 | -1.06865 | finngen-R9-E4-THYROIDITAUTOIM | 0.030007 | 3.10E-05 | 0.066481 | 22873.63 |
| 40 | rs4776793 | T | C | 321192 | 0.064195 | 0.267873 | finngen-R9-E4-THYROIDITAUTOIM | 0.327436 | 3.01E-05 | 0.031604 | 10482.31 |
| 41 | rs112331534 | C | T | 321192 | 0.141005 | 0.633624 | finngen-R9-E4-THYROIDITAUTOIM | 0.035549 | 7.00E-06 | 0.02753 | 9092.566 |
| 42 | rs73284905 | A | G | 321192 | 0.070683 | 0.30827 | finngen-R9-E4-THYROIDITAUTOIM | 0.227077 | 1.29E-05 | 0.033358 | 11084.05 |
| 43 | rs150596578 | T | C | 321192 | 0.195784 | 0.884001 | finngen-R9-E4-THYROIDITAUTOIM | 0.015873 | 6.33E-06 | 0.024414 | 8037.894 |
| 44 | rs143412024 | A | G | 321192 | 0.206271 | 0.87 | finngen-R9-E4-THYROIDITAUTOIM | 0.013827 | 2.47E-05 | 0.020642 | 6769.907 |
| 45 | rs62075181 | T | C | 321192 | 0.107706 | 0.442435 | finngen-R9-E4-THYROIDITAUTOIM | 0.071876 | 3.99E-05 | 0.026117 | 8613.364 |
| 46 | rs8088539 | A | G | 321192 | 0.084682 | -0.39191 | finngen-R9-E4-THYROIDITAUTOIM | 0.869113 | 3.69E-06 | 0.034944 | 11630.08 |
| 47 | rs532558 | C | T | 321192 | 0.081609 | -0.35969 | finngen-R9-E4-THYROIDITAUTOIM | 0.856798 | 1.05E-05 | 0.031748 | 10531.56 |
| 48 | rs8096446 | C | T | 321192 | 0.091908 | -0.37934 | finngen-R9-E4-THYROIDITAUTOIM | 0.162379 | 3.67E-05 | 0.039145 | 13085.05 |
| 49 | rs7242091 | G | A | 321192 | 0.064947 | -0.27487 | finngen-R9-E4-THYROIDITAUTOIM | 0.663205 | 2.31E-05 | 0.033752 | 11219.45 |
| 50 | rs111974140 | C | T | 321192 | 0.065547 | -0.27727 | finngen-R9-E4-THYROIDITAUTOIM | 0.38564 | 2.34E-05 | 0.036427 | 12142.44 |
| 51 | rs214825 | C | T | 321192 | 0.105748 | -0.43981 | finngen-R9-E4-THYROIDITAUTOIM | 0.921172 | 3.20E-05 | 0.028092 | 9283.552 |
| 52 | rs3746756 | T | C | 321192 | 0.098602 | -0.41759 | finngen-R9-E4-THYROIDITAUTOIM | 0.140346 | 2.28E-05 | 0.042079 | 14108.94 |
| 53 | rs9653729 | C | T | 321192 | 0.06808 | 0.282481 | finngen-R9-E4-THYROIDITAUTOIM | 0.263488 | 3.34E-05 | 0.030971 | 10265.36 |

SNP, single nucleotide polymorphism; EA, effect allele; OA, other allele; EAF, effect allele frequency; SE, standard error; AIT, autoimmune thyroiditis.

**Supplementary Table S1.2** Characteristics of instrumental variables for IBD.

|  | **SNP** | **EA** | **OA** | **Samplesize** | **SE** | **β** | **id.exposure** | **EAF** | ***p* value** | **R^2^** | **F - statistic** |
| --- | --- | --- | --- | --- | --- | --- | --- | --- | --- | --- | --- |
| 1 | rs12755372 | C | A | 377277 | 0.019331 | -0.11737 | finngen-R9-K11-IBD-STRICT | 0.229429 | 1.27E-09 | 0.004871 | 1846.615 |
| 2 | rs10737481 | G | T | 377277 | 0.016387 | 0.144709 | finngen-R9-K11-IBD-STRICT | 0.544803 | 1.04E-18 | 0.010386 | 3959.609 |
| 3 | rs9988642 | C | T | 377277 | 0.044693 | -0.45969 | finngen-R9-K11-IBD-STRICT | 0.029576 | 8.19E-25 | 0.01213 | 4632.572 |
| 4 | rs376365394 | T | C | 377277 | 0.039053 | 0.245757 | finngen-R9-K11-IBD-STRICT | 0.050903 | 3.11E-10 | 0.005836 | 2214.61 |
| 5 | rs191615076 | A | C | 377277 | 0.039372 | 0.246276 | finngen-R9-K11-IBD-STRICT | 0.050813 | 3.97E-10 | 0.005851 | 2220.285 |
| 6 | rs12132298 | C | T | 377277 | 0.020651 | -0.16281 | finngen-R9-K11-IBD-STRICT | 0.186365 | 3.18E-15 | 0.008038 | 3057.191 |
| 7 | rs67927699 | C | G | 377277 | 0.016812 | 0.102968 | finngen-R9-K11-IBD-STRICT | 0.39439 | 9.09E-10 | 0.005065 | 1920.51 |
| 8 | rs1882597 | C | A | 377277 | 0.036469 | 0.203803 | finngen-R9-K11-IBD-STRICT | 0.056941 | 2.29E-08 | 0.004461 | 1690.49 |
| 9 | rs10931828 | T | C | 377277 | 0.016588 | -0.09523 | finngen-R9-K11-IBD-STRICT | 0.417992 | 9.43E-09 | 0.004412 | 1671.923 |
| 10 | rs4676410 | A | G | 377277 | 0.018097 | 0.165787 | finngen-R9-K11-IBD-STRICT | 0.304322 | 5.15E-20 | 0.011638 | 4442.367 |
| 11 | rs895123 | G | C | 377277 | 0.020129 | 0.142454 | finngen-R9-K11-IBD-STRICT | 0.216775 | 1.47E-12 | 0.006891 | 2617.798 |
| 12 | rs13165038 | C | T | 377277 | 0.018048 | -0.13114 | finngen-R9-K11-IBD-STRICT | 0.285334 | 3.69E-13 | 0.007014 | 2665.008 |
| 13 | rs6556416 | C | A | 377277 | 0.019169 | 0.112882 | finngen-R9-K11-IBD-STRICT | 0.763968 | 3.89E-09 | 0.004595 | 1741.742 |
| 14 | rs12536069 | C | T | 377277 | 0.030335 | 0.252018 | finngen-R9-K11-IBD-STRICT | 0.085902 | 9.74E-17 | 0.009974 | 3801.034 |
| 15 | rs56893428 | T | C | 377277 | 0.016465 | 0.091268 | finngen-R9-K11-IBD-STRICT | 0.474613 | 2.97E-08 | 0.004154 | 1573.809 |
| 16 | rs10807943 | C | T | 377277 | 0.030745 | -0.34122 | finngen-R9-K11-IBD-STRICT | 0.913986 | 1.28E-28 | 0.018307 | 7035.584 |
| 17 | rs181316459 | C | G | 377277 | 0.032675 | 0.61638 | finngen-R9-K11-IBD-STRICT | 0.082917 | 2.25E-79 | 0.05778 | 23135.86 |
| 18 | rs142695953 | A | C | 377277 | 0.020411 | 0.18025 | finngen-R9-K11-IBD-STRICT | 0.214461 | 1.04E-18 | 0.010947 | 4175.756 |
| 19 | rs11771806 | T | C | 377277 | 0.024712 | 0.135166 | finngen-R9-K11-IBD-STRICT | 0.131825 | 4.51E-08 | 0.004182 | 1584.338 |
| 20 | rs4730275 | T | G | 377277 | 0.017627 | -0.11374 | finngen-R9-K11-IBD-STRICT | 0.308887 | 1.10E-10 | 0.005523 | 2095.223 |
| 21 | rs2149560 | T | G | 377277 | 0.016549 | 0.12233 | finngen-R9-K11-IBD-STRICT | 0.582761 | 1.44E-13 | 0.007277 | 2765.677 |
| 22 | rs4372078 | G | T | 377277 | 0.018993 | 0.121229 | finngen-R9-K11-IBD-STRICT | 0.760985 | 1.74E-10 | 0.005346 | 2027.825 |
| 23 | rs1986500 | A | G | 377277 | 0.016541 | 0.099924 | finngen-R9-K11-IBD-STRICT | 0.46704 | 1.53E-09 | 0.004971 | 1884.7 |
| 24 | rs117115824 | T | A | 377277 | 0.029779 | -0.16683 | finngen-R9-K11-IBD-STRICT | 0.089955 | 2.12E-08 | 0.004557 | 1726.952 |
| 25 | rs3736162 | C | G | 377277 | 0.018467 | -0.10841 | finngen-R9-K11-IBD-STRICT | 0.260731 | 4.34E-09 | 0.004531 | 1717.094 |
| 26 | rs4807543 | T | G | 377277 | 0.043471 | 0.23941 | finngen-R9-K11-IBD-STRICT | 0.039933 | 3.64E-08 | 0.004395 | 1665.398 |
| 27 | rs6017342 | C | A | 377277 | 0.016616 | 0.122771 | finngen-R9-K11-IBD-STRICT | 0.588598 | 1.48E-13 | 0.0073 | 2774.257 |
| 28 | rs3827023 | T | C | 377277 | 0.021572 | -0.14229 | finngen-R9-K11-IBD-STRICT | 0.167734 | 4.22E-11 | 0.005653 | 2144.685 |
| 29 | rs2836883 | A | G | 377277 | 0.019459 | -0.14097 | finngen-R9-K11-IBD-STRICT | 0.221841 | 4.34E-13 | 0.006861 | 2606.515 |
| 30 | rs9607629 | G | A | 377277 | 0.026026 | -0.17825 | finngen-R9-K11-IBD-STRICT | 0.106011 | 7.43E-12 | 0.006023 | 2285.984 |
| 31 | rs9617090 | T | C | 377277 | 0.017137 | -0.12894 | finngen-R9-K11-IBD-STRICT | 0.343388 | 5.30E-14 | 0.007498 | 2850.007 |

SNP, single nucleotide polymorphism; EA, effect allele; OA, other allele; EAF, effect allele frequency; SE, standard error; IBD, inflammatory bowel disease.

**Supplementary Table S1.3** Characteristics of instrumental variables for UC.

|  | **SNP** | **EA** | **OA** | **Samplesize** | **SE** | **β** | **id.exposure** | **EAF** | ***p* value** | **R^2^** | **F - statistic** |
| --- | --- | --- | --- | --- | --- | --- | --- | --- | --- | --- | --- |
| 1 | rs12736494 | A | G | 376564 | 0.023245 | -0.13767 | finngen-R9-K11-UC-STRICT2 | 0.264236 | 3.17E-09 | 0.007369 | 2800.813 |
| 2 | rs10799837 | A | G | 376564 | 0.020212 | -0.11875 | finngen-R9-K11-UC-STRICT2 | 0.506968 | 4.22E-09 | 0.00705 | 2678.494 |
| 3 | rs10737481 | G | T | 376564 | 0.020053 | 0.177217 | finngen-R9-K11-UC-STRICT2 | 0.509662 | 9.78E-19 | 0.015697 | 6016.554 |
| 4 | rs11209026 | A | G | 376564 | 0.053654 | -0.38229 | finngen-R9-K11-UC-STRICT2 | 0.045692 | 1.04E-12 | 0.012745 | 4870.534 |
| 5 | rs12132298 | C | T | 376564 | 0.025256 | -0.15483 | finngen-R9-K11-UC-STRICT2 | 0.213061 | 8.76E-10 | 0.008039 | 3057.493 |
| 6 | rs3024495 | T | C | 376564 | 0.025977 | 0.262195 | finngen-R9-K11-UC-STRICT2 | 0.157263 | 5.91E-24 | 0.018222 | 7002.324 |
| 7 | rs1878668 | G | T | 376564 | 0.0206 | 0.119883 | finngen-R9-K11-UC-STRICT2 | 0.595569 | 5.90E-09 | 0.006923 | 2630.25 |
| 8 | rs13024106 | A | G | 376564 | 0.020206 | -0.1159 | finngen-R9-K11-UC-STRICT2 | 0.583289 | 9.69E-09 | 0.00653 | 2479.899 |
| 9 | rs34236350 | T | C | 376564 | 0.022135 | 0.17698 | finngen-R9-K11-UC-STRICT2 | 0.267419 | 1.29E-15 | 0.012272 | 4687.563 |
| 10 | rs13165038 | C | T | 376564 | 0.022105 | -0.13335 | finngen-R9-K11-UC-STRICT2 | 0.312207 | 1.61E-09 | 0.007637 | 2903.336 |
| 11 | rs12536069 | C | T | 376564 | 0.037218 | 0.245799 | finngen-R9-K11-UC-STRICT2 | 0.068398 | 4.00E-11 | 0.0077 | 2927.394 |
| 12 | rs10807943 | C | T | 376564 | 0.037902 | -0.31354 | finngen-R9-K11-UC-STRICT2 | 0.936436 | 1.31E-16 | 0.011703 | 4467.472 |
| 13 | rs181316459 | C | G | 376564 | 0.040174 | 0.597776 | finngen-R9-K11-UC-STRICT2 | 0.047393 | 4.46E-50 | 0.032265 | 12578.83 |
| 14 | rs142695953 | A | C | 376564 | 0.024897 | 0.19257 | finngen-R9-K11-UC-STRICT2 | 0.1863 | 1.04E-14 | 0.011243 | 4289.957 |
| 15 | rs6967335 | A | C | 376564 | 0.020383 | -0.15636 | finngen-R9-K11-UC-STRICT2 | 0.43172 | 1.70E-14 | 0.011996 | 4580.842 |
| 16 | rs7865719 | G | A | 376564 | 0.020261 | 0.123757 | finngen-R9-K11-UC-STRICT2 | 0.55643 | 1.01E-09 | 0.00756 | 2874.062 |
| 17 | rs4263839 | G | A | 376564 | 0.022366 | 0.12357 | finngen-R9-K11-UC-STRICT2 | 0.706524 | 3.30E-08 | 0.006332 | 2404.21 |
| 18 | rs10748781 | A | C | 376564 | 0.020789 | -0.15627 | finngen-R9-K11-UC-STRICT2 | 0.652344 | 5.60E-14 | 0.011076 | 4225.633 |
| 19 | rs7930763 | A | G | 376564 | 0.020125 | 0.133479 | finngen-R9-K11-UC-STRICT2 | 0.439905 | 3.30E-11 | 0.00878 | 3341.676 |
| 20 | rs56086041 | G | T | 376564 | 0.029056 | -0.17927 | finngen-R9-K11-UC-STRICT2 | 0.164664 | 6.84E-10 | 0.008841 | 3365.118 |
| 21 | rs6017342 | C | A | 376564 | 0.020348 | 0.149349 | finngen-R9-K11-UC-STRICT2 | 0.559104 | 2.14E-13 | 0.010997 | 4194.92 |
| 22 | rs6089926 | T | C | 376564 | 0.025723 | -0.16264 | finngen-R9-K11-UC-STRICT2 | 0.207998 | 2.57E-10 | 0.008715 | 3316.921 |
| 23 | rs4817986 | T | G | 376564 | 0.023884 | -0.15786 | finngen-R9-K11-UC-STRICT2 | 0.247267 | 3.85E-11 | 0.009277 | 3532.583 |
| 24 | rs9607629 | G | A | 376564 | 0.032047 | -0.20304 | finngen-R9-K11-UC-STRICT2 | 0.125017 | 2.36E-10 | 0.009019 | 3433.571 |
| 25 | rs9617090 | T | C | 376564 | 0.021029 | -0.16385 | finngen-R9-K11-UC-STRICT2 | 0.372128 | 6.60E-15 | 0.012546 | 4793.278 |

SNP, single nucleotide polymorphism; EA, effect allele; OA, other allele; EAF, effect allele frequency; SE, standard error; UC, ulcerative colitis.

**Supplementary Table S1.4** Characteristics of instrumental variables for CD.

|  | **SNP** | **EA** | **OA** | **Samplesize** | **SE** | **β** | **id.exposure** | **EAF** | ***p* value** | **R^2^** | **F - statistic** |
| --- | --- | --- | --- | --- | --- | --- | --- | --- | --- | --- | --- |
| 1 | rs12097131 | G | C | 377277 | 0.011018 | 0.053592 | finngen-R9-RX-CROHN-1STLINE | 0.075863 | 1.15E-06 | 0.000403 | 151.9925 |
| 2 | rs12049158 | C | G | 377277 | 0.007943 | 0.037406 | finngen-R9-RX-CROHN-1STLINE | 0.1585 | 2.48E-06 | 0.000373 | 140.8722 |
| 3 | rs35300988 | A | C | 377277 | 0.021537 | 0.100103 | finngen-R9-RX-CROHN-1STLINE | 0.018413 | 3.35E-06 | 0.000362 | 136.7069 |
| 4 | rs557541 | T | C | 377277 | 0.005884 | 0.028117 | finngen-R9-RX-CROHN-1STLINE | 0.582147 | 1.77E-06 | 0.000385 | 145.1639 |
| 5 | rs12131154 | A | G | 377277 | 0.007279 | -0.03695 | finngen-R9-RX-CROHN-1STLINE | 0.202388 | 3.85E-07 | 0.000441 | 166.3765 |
| 6 | rs79004809 | A | G | 377277 | 0.008782 | 0.040963 | finngen-R9-RX-CROHN-1STLINE | 0.124437 | 3.09E-06 | 0.000366 | 137.9963 |
| 7 | rs4657041 | C | T | 377277 | 0.005788 | -0.03075 | finngen-R9-RX-CROHN-1STLINE | 0.504441 | 1.08E-07 | 0.000473 | 178.483 |
| 8 | rs1917534 | A | G | 377277 | 0.006061 | -0.03187 | finngen-R9-RX-CROHN-1STLINE | 0.361129 | 1.46E-07 | 0.000469 | 176.8481 |
| 9 | rs1052238 | C | T | 377277 | 0.005845 | 0.03004 | finngen-R9-RX-CROHN-1STLINE | 0.54112 | 2.76E-07 | 0.000448 | 169.1468 |
| 10 | rs1182871 | A | G | 377277 | 0.005942 | 0.027484 | finngen-R9-RX-CROHN-1STLINE | 0.403987 | 3.73E-06 | 0.000364 | 137.2899 |
| 11 | rs7576788 | C | T | 377277 | 0.005858 | 0.030032 | finngen-R9-RX-CROHN-1STLINE | 0.430813 | 2.95E-07 | 0.000442 | 166.9523 |
| 12 | rs10167906 | C | A | 377277 | 0.006358 | -0.03055 | finngen-R9-RX-CROHN-1STLINE | 0.297404 | 1.55E-06 | 0.00039 | 147.1861 |
| 13 | rs56084808 | T | C | 377277 | 0.011913 | -0.05621 | finngen-R9-RX-CROHN-1STLINE | 0.064776 | 2.38E-06 | 0.000383 | 144.4595 |
| 14 | rs77791527 | G | A | 377277 | 0.01702 | -0.09057 | finngen-R9-RX-CROHN-1STLINE | 0.030742 | 1.03E-07 | 0.000489 | 184.5133 |
| 15 | rs7605284 | G | T | 377277 | 0.00581 | -0.02873 | finngen-R9-RX-CROHN-1STLINE | 0.519966 | 7.64E-07 | 0.000412 | 155.4709 |
| 16 | rs79918045 | A | G | 377277 | 0.009409 | 0.06783 | finngen-R9-RX-CROHN-1STLINE | 0.10449 | 5.62E-13 | 0.000861 | 325.1264 |
| 17 | rs1271066 | A | G | 377277 | 0.006963 | 0.040851 | finngen-R9-RX-CROHN-1STLINE | 0.77367 | 4.44E-09 | 0.000584 | 220.6191 |
| 18 | rs62175521 | G | A | 377277 | 0.013095 | -0.06043 | finngen-R9-RX-CROHN-1STLINE | 0.052866 | 3.93E-06 | 0.000366 | 138.03 |
| 19 | rs150886680 | T | C | 377277 | 0.021648 | -0.10617 | finngen-R9-RX-CROHN-1STLINE | 0.01953 | 9.37E-07 | 0.000432 | 162.9464 |
| 20 | rs7590672 | C | T | 377277 | 0.013332 | -0.08179 | finngen-R9-RX-CROHN-1STLINE | 0.951032 | 8.54E-10 | 0.000623 | 235.2009 |
| 21 | rs7630905 | A | G | 377277 | 0.005857 | -0.03116 | finngen-R9-RX-CROHN-1STLINE | 0.562828 | 1.04E-07 | 0.000478 | 180.373 |
| 22 | rs34227248 | A | T | 377277 | 0.007059 | 0.036764 | finngen-R9-RX-CROHN-1STLINE | 0.212173 | 1.91E-07 | 0.000452 | 170.545 |
| 23 | rs1969066 | A | G | 377277 | 0.006897 | 0.034076 | finngen-R9-RX-CROHN-1STLINE | 0.227155 | 7.79E-07 | 0.000408 | 153.8809 |
| 24 | rs7609646 | A | G | 377277 | 0.008778 | 0.043508 | finngen-R9-RX-CROHN-1STLINE | 0.12545 | 7.18E-07 | 0.000415 | 156.7668 |
| 25 | rs66654254 | A | G | 377277 | 0.005905 | -0.0301 | finngen-R9-RX-CROHN-1STLINE | 0.434797 | 3.43E-07 | 0.000445 | 168.1083 |
| 26 | rs12645439 | A | C | 377277 | 0.00611 | -0.02903 | finngen-R9-RX-CROHN-1STLINE | 0.35606 | 2.01E-06 | 0.000387 | 145.9013 |
| 27 | rs34696890 | T | C | 377277 | 0.006863 | -0.03217 | finngen-R9-RX-CROHN-1STLINE | 0.23439 | 2.78E-06 | 0.000371 | 140.1438 |
| 28 | rs11097241 | T | C | 377277 | 0.006326 | 0.030581 | finngen-R9-RX-CROHN-1STLINE | 0.698904 | 1.34E-06 | 0.000394 | 148.5503 |

SNP, single nucleotide polymorphism; EA, effect allele; OA, other allele; EAF, effect allele frequency; SE, standard error; CD, Crohn's disease.

**Supplementary Table S2.1** SNPs from GWAS on AIT and IBD.

|  |  | | | | **Exposure (AIT)** | | |  | | **Outcome (IBD)** | | | | |
| --- | --- | --- | --- | --- | --- | --- | --- | --- | --- | --- | --- | --- | --- | --- |
|  | **SNP** | **EA** | **OA** | **β** | | **SE** | ***p* value** |  | **Case** | | **Control** | **β** | **SE** | ***p* value** |
| 1 | rs1025256 | G | A | 0.314269 | | 0.065123 | 1.39E-06 |  | 7,625 | | 369,652 | 0.0137733 | 0.0178793 | 0.441093 |
| 2 | rs10865180 | A | G | 0.329956 | | 0.079889 | 3.62E-05 |  | 7,625 | | 369,652 | 0.0185215 | 0.022646 | 0.413432 |
| 3 | rs11061760 | C | T | 0.30745 | | 0.075124 | 4.27E-05 |  | 7,625 | | 369,652 | -0.0162891 | 0.0211525 | 0.441253 |
| 4 | rs11192013 | C | T | -0.291 | | 0.069748 | 3.02E-05 |  | 7,625 | | 369,652 | 0.0301367 | 0.0176804 | 0.0882816 |
| 5 | rs111974140 | C | T | -0.27727 | | 0.065547 | 2.34E-05 |  | 7,625 | | 369,652 | -0.000411332 | 0.0168704 | 0.980548 |
| 6 | rs112241357 | A | G | 0.574445 | | 0.138244 | 3.25E-05 |  | 7,625 | | 369,652 | -0.00953143 | 0.0442846 | 0.829587 |
| 7 | rs112331534 | C | T | 0.633624 | | 0.141005 | 7.00E-06 |  | 7,625 | | 369,652 | -0.07843 | 0.0449415 | 0.080958 |
| 8 | rs114644112 | G | A | 0.753603 | | 0.177922 | 2.28E-05 |  | 7,625 | | 369,652 | -0.0428901 | 0.0588581 | 0.466183 |
| 9 | rs115513802 | C | T | 0.588563 | | 0.144354 | 4.56E-05 |  | 7,625 | | 369,652 | 0.0304379 | 0.0459205 | 0.507435 |
| 10 | rs11706033 | T | C | -0.38873 | | 0.091815 | 2.30E-05 |  | 7,625 | | 369,652 | -0.00406124 | 0.0222242 | 0.855003 |
| 11 | rs117206250 | T | A | 1.14957 | | 0.274268 | 2.77E-05 |  | 7,625 | | 369,652 | -0.168387 | 0.107224 | 0.116317 |
| 12 | rs117944015 | T | C | 1.44667 | | 0.337467 | 1.81E-05 |  | 7,625 | | 369,652 | -0.115759 | 0.152342 | 0.447339 |
| 13 | rs118162083 | G | A | 1.18895 | | 0.287166 | 3.47E-05 |  | 7,625 | | 369,652 | 0.241766 | 0.110967 | 0.0293528 |
| 14 | rs11837712 | C | G | 0.795142 | | 0.179719 | 9.67E-06 |  | 7,625 | | 369,652 | -0.0846882 | 0.0602342 | 0.159729 |
| 15 | rs12589558 | C | T | 0.319573 | | 0.074585 | 1.83E-05 |  | 7,625 | | 369,652 | -0.0285659 | 0.0207888 | 0.169412 |
| 16 | rs138602074 | T | C | -0.88033 | | 0.212512 | 3.44E-05 |  | 7,625 | | 369,652 | 0.0712402 | 0.0435936 | 0.102219 |
| 17 | rs143412024 | A | G | 0.87 | | 0.206271 | 2.47E-05 |  | 7,625 | | 369,652 | -0.0212681 | 0.0708893 | 0.764163 |
| 18 | rs144513952 | A | G | 0.667098 | | 0.158488 | 2.56E-05 |  | 7,625 | | 369,652 | 0.00419473 | 0.0512343 | 0.934747 |
| 19 | rs145519624 | C | A | 0.613155 | | 0.1464 | 2.81E-05 |  | 7,625 | | 369,652 | 0.00557756 | 0.0466448 | 0.90482 |
| 20 | rs149021369 | T | C | -1.06865 | | 0.256528 | 3.10E-05 |  | 7,625 | | 369,652 | 0.034777 | 0.0485685 | 0.473966 |
| 21 | rs150596578 | T | C | 0.884001 | | 0.195784 | 6.33E-06 |  | 7,625 | | 369,652 | 0.124909 | 0.0681633 | 0.0668775 |
| 22 | rs150879737 | T | C | 1.15009 | | 0.264898 | 1.41E-05 |  | 7,625 | | 369,652 | -0.032409 | 0.106472 | 0.760831 |
| 23 | rs1604966 | C | G | 0.342512 | | 0.066388 | 2.48E-07 |  | 7,625 | | 369,652 | 0.0068446 | 0.0184189 | 0.710186 |
| 24 | rs16967767 | T | C | 1.19627 | | 0.29416 | 4.77E-05 |  | 7,625 | | 369,652 | 0.0438973 | 0.120148 | 0.714842 |
| 25 | rs1800009 | C | T | 0.272537 | | 0.063752 | 1.91E-05 |  | 7,625 | | 369,652 | 0.0022322 | 0.0172036 | 0.896762 |
| 26 | rs214825 | C | T | -0.43981 | | 0.105748 | 3.20E-05 |  | 7,625 | | 369,652 | 0.00531095 | 0.0311414 | 0.864583 |
| 27 | rs3116997 | A | G | -0.48694 | | 0.090937 | 8.57E-08 |  | 7,625 | | 369,652 | 0.00812458 | 0.0217553 | 0.708812 |
| 28 | rs35173692 | A | T | -1.06458 | | 0.257188 | 3.48E-05 |  | 7,625 | | 369,652 | 0.0460026 | 0.0513253 | 0.370096 |
| 29 | rs35529104 | T | C | 1.07077 | | 0.261141 | 4.13E-05 |  | 7,625 | | 369,652 | 0.162699 | 0.100808 | 0.106536 |
| 30 | rs3746756 | T | C | -0.41759 | | 0.098602 | 2.28E-05 |  | 7,625 | | 369,652 | 0.0272794 | 0.0236725 | 0.249171 |
| 31 | rs4776793 | T | C | 0.267873 | | 0.064195 | 3.01E-05 |  | 7,625 | | 369,652 | -0.0140208 | 0.0174781 | 0.422441 |
| 32 | rs4947750 | C | T | 0.667293 | | 0.160791 | 3.32E-05 |  | 7,625 | | 369,652 | 0.00288751 | 0.0517966 | 0.955543 |
| 33 | rs532558 | C | T | -0.35969 | | 0.081609 | 1.05E-05 |  | 7,625 | | 369,652 | 5.59E-05 | 0.0234952 | 0.998103 |
| 34 | rs55906846 | G | C | 0.509594 | | 0.105867 | 1.48E-06 |  | 7,625 | | 369,652 | 0.00928415 | 0.0322751 | 0.77361 |
| 35 | rs56224189 | T | G | 0.632792 | | 0.150782 | 2.71E-05 |  | 7,625 | | 369,652 | -0.059492 | 0.0479231 | 0.214456 |
| 36 | rs61870853 | C | A | 1.08581 | | 0.267453 | 4.91E-05 |  | 7,625 | | 369,652 | 0.0404875 | 0.0998959 | 0.685259 |
| 37 | rs62075181 | T | C | 0.442435 | | 0.107706 | 3.99E-05 |  | 7,625 | | 369,652 | 0.0364417 | 0.031857 | 0.252659 |
| 38 | rs6785758 | A | G | 0.364884 | | 0.081055 | 6.74E-06 |  | 7,625 | | 369,652 | -0.00615509 | 0.0234394 | 0.792861 |
| 39 | rs7242091 | G | A | -0.27487 | | 0.064947 | 2.31E-05 |  | 7,625 | | 369,652 | 0.00200805 | 0.0175841 | 0.909082 |
| 40 | rs72682840 | T | C | -0.62632 | | 0.151106 | 3.40E-05 |  | 7,625 | | 369,652 | -0.0391987 | 0.033436 | 0.241057 |
| 41 | rs73237426 | T | A | 0.462271 | | 0.106278 | 1.36E-05 |  | 7,625 | | 369,652 | 0.0282228 | 0.0319644 | 0.377266 |
| 42 | rs73284905 | A | G | 0.30827 | | 0.070683 | 1.29E-05 |  | 7,625 | | 369,652 | 0.00650693 | 0.0196246 | 0.740215 |
| 43 | rs76196487 | G | A | 0.848037 | | 0.200174 | 2.27E-05 |  | 7,625 | | 369,652 | 0.0141492 | 0.0710062 | 0.842054 |
| 44 | rs7703397 | C | A | 0.311465 | | 0.070007 | 8.62E-06 |  | 7,625 | | 369,652 | -0.0293017 | 0.0177289 | 0.0983785 |
| 45 | rs77880724 | T | A | 0.39086 | | 0.092581 | 2.42E-05 |  | 7,625 | | 369,652 | 0.0727492 | 0.0266278 | 0.00629376 |
| 46 | rs79339719 | A | C | 0.606856 | | 0.144127 | 2.55E-05 |  | 7,625 | | 369,652 | -0.0378243 | 0.0447238 | 0.397704 |
| 47 | rs80101832 | G | A | -0.29745 | | 0.071367 | 3.07E-05 |  | 7,625 | | 369,652 | -0.00050159 | 0.0179978 | 0.977766 |
| 48 | rs8088539 | A | G | -0.39191 | | 0.084682 | 3.69E-06 |  | 7,625 | | 369,652 | 0.00488116 | 0.0244471 | 0.841745 |
| 49 | rs8096446 | C | T | -0.37934 | | 0.091908 | 3.67E-05 |  | 7,625 | | 369,652 | 0.00496982 | 0.0223588 | 0.824099 |
| 50 | rs9653729 | C | T | 0.282481 | | 0.06808 | 3.34E-05 |  | 7,625 | | 369,652 | 0.0223345 | 0.0186817 | 0.23188 |

SNP, single nucleotide polymorphism; EA, effect allele; OA, other allele; SE, standard error; AIT, autoimmune thyroiditis; IBD, inflammatory bowel disease.

**Supplementary Table S2.2** SNPs from GWAS on AIT and UC.

|  |  | | | | **Exposure (AIT)** | | |  | | **Outcome (UC)** | | | | |
| --- | --- | --- | --- | --- | --- | --- | --- | --- | --- | --- | --- | --- | --- | --- |
|  | **SNP** | **EA** | **OA** | **β** | | **SE** | ***p* value** |  | **Case** | | **Control** | **β** | **SE** | ***p* value** |
| 1 | rs1025256 | G | A | 0.314269 | | 0.065123 | 1.39E-06 |  | 5,034 | | 371,530 | 0.01022 | 0.021908 | 0.640864 |
| 2 | rs10865180 | A | G | 0.329956 | | 0.079889 | 3.62E-05 |  | 5,034 | | 371,530 | 0.015695 | 0.027739 | 0.571513 |
| 3 | rs11061760 | C | T | 0.30745 | | 0.075124 | 4.27E-05 |  | 5,034 | | 371,530 | -0.01813 | 0.025914 | 0.484046 |
| 4 | rs11192013 | C | T | -0.291 | | 0.069748 | 3.02E-05 |  | 5,034 | | 371,530 | 0.011297 | 0.021661 | 0.601998 |
| 5 | rs111974140 | C | T | -0.27727 | | 0.065547 | 2.34E-05 |  | 5,034 | | 371,530 | 0.009539 | 0.020667 | 0.644417 |
| 6 | rs112241357 | A | G | 0.574445 | | 0.138244 | 3.25E-05 |  | 5,034 | | 371,530 | -0.03663 | 0.054105 | 0.498453 |
| 7 | rs112331534 | C | T | 0.633624 | | 0.141005 | 7.00E-06 |  | 5,034 | | 371,530 | -0.11219 | 0.055126 | 0.041837 |
| 8 | rs114644112 | G | A | 0.753603 | | 0.177922 | 2.28E-05 |  | 5,034 | | 371,530 | -0.1241 | 0.072059 | 0.085022 |
| 9 | rs115513802 | C | T | 0.588563 | | 0.144354 | 4.56E-05 |  | 5,034 | | 371,530 | 0.092265 | 0.05633 | 0.101437 |
| 10 | rs11706033 | T | C | -0.38873 | | 0.091815 | 2.30E-05 |  | 5,034 | | 371,530 | -0.01865 | 0.027214 | 0.493048 |
| 11 | rs117206250 | T | A | 1.14957 | | 0.274268 | 2.77E-05 |  | 5,034 | | 371,530 | -0.15811 | 0.131767 | 0.230162 |
| 12 | rs117944015 | T | C | 1.44667 | | 0.337467 | 1.81E-05 |  | 5,034 | | 371,530 | -0.20093 | 0.18644 | 0.281165 |
| 13 | rs118162083 | G | A | 1.18895 | | 0.287166 | 3.47E-05 |  | 5,034 | | 371,530 | 0.236835 | 0.136515 | 0.082764 |
| 14 | rs11837712 | C | G | 0.795142 | | 0.179719 | 9.67E-06 |  | 5,034 | | 371,530 | -0.08362 | 0.073698 | 0.256505 |
| 15 | rs12589558 | C | T | 0.319573 | | 0.074585 | 1.83E-05 |  | 5,034 | | 371,530 | -0.04555 | 0.025481 | 0.073816 |
| 16 | rs138602074 | T | C | -0.88033 | | 0.212512 | 3.44E-05 |  | 5,034 | | 371,530 | 0.074551 | 0.053345 | 0.162251 |
| 17 | rs143412024 | A | G | 0.87 | | 0.206271 | 2.47E-05 |  | 5,034 | | 371,530 | -0.03398 | 0.086797 | 0.695445 |
| 18 | rs144513952 | A | G | 0.667098 | | 0.158488 | 2.56E-05 |  | 5,034 | | 371,530 | 0.034141 | 0.06299 | 0.587814 |
| 19 | rs145519624 | C | A | 0.613155 | | 0.1464 | 2.81E-05 |  | 5,034 | | 371,530 | 0.049469 | 0.057162 | 0.386808 |
| 20 | rs149021369 | T | C | -1.06865 | | 0.256528 | 3.10E-05 |  | 5,034 | | 371,530 | 0.025318 | 0.059434 | 0.670122 |
| 21 | rs150596578 | T | C | 0.884001 | | 0.195784 | 6.33E-06 |  | 5,034 | | 371,530 | 0.09871 | 0.083701 | 0.238269 |
| 22 | rs150879737 | T | C | 1.15009 | | 0.264898 | 1.41E-05 |  | 5,034 | | 371,530 | -0.04562 | 0.130383 | 0.726397 |
| 23 | rs1604966 | C | G | 0.342512 | | 0.066388 | 2.48E-07 |  | 5,034 | | 371,530 | 0.040039 | 0.022564 | 0.075994 |
| 24 | rs16967767 | T | C | 1.19627 | | 0.29416 | 4.77E-05 |  | 5,034 | | 371,530 | -0.03923 | 0.147093 | 0.789702 |
| 25 | rs1800009 | C | T | 0.272537 | | 0.063752 | 1.91E-05 |  | 5,034 | | 371,530 | 0.007625 | 0.021074 | 0.717477 |
| 26 | rs214825 | C | T | -0.43981 | | 0.105748 | 3.20E-05 |  | 5,034 | | 371,530 | 0.012875 | 0.038092 | 0.735374 |
| 27 | rs2707275 | T | C | 0.270325 | | 0.062625 | 1.58E-05 |  | 5,034 | | 371,530 | 0.041085 | 0.020356 | 0.043553 |
| 28 | rs3116997 | A | G | -0.48694 | | 0.090937 | 8.57E-08 |  | 5,034 | | 371,530 | -0.02834 | 0.026649 | 0.28754 |
| 29 | rs35173692 | A | T | -1.06458 | | 0.257188 | 3.48E-05 |  | 5,034 | | 371,530 | 0.048032 | 0.062768 | 0.444136 |
| 30 | rs35529104 | T | C | 1.07077 | | 0.261141 | 4.13E-05 |  | 5,034 | | 371,530 | 0.135367 | 0.123542 | 0.273205 |
| 31 | rs3746756 | T | C | -0.41759 | | 0.098602 | 2.28E-05 |  | 5,034 | | 371,530 | -0.00017 | 0.029028 | 0.995413 |
| 32 | rs4776793 | T | C | 0.267873 | | 0.064195 | 3.01E-05 |  | 5,034 | | 371,530 | 0.004775 | 0.021412 | 0.823539 |
| 33 | rs4947750 | C | T | 0.667293 | | 0.160791 | 3.32E-05 |  | 5,034 | | 371,530 | 0.004985 | 0.063423 | 0.93735 |
| 34 | rs532558 | C | T | -0.35969 | | 0.081609 | 1.05E-05 |  | 5,034 | | 371,530 | 0.004849 | 0.028774 | 0.866181 |
| 35 | rs55906846 | G | C | 0.509594 | | 0.105867 | 1.48E-06 |  | 5,034 | | 371,530 | 0.006829 | 0.039521 | 0.862809 |
| 36 | rs56224189 | T | G | 0.632792 | | 0.150782 | 2.71E-05 |  | 5,034 | | 371,530 | -0.02804 | 0.05873 | 0.633015 |
| 37 | rs61870853 | C | A | 1.08581 | | 0.267453 | 4.91E-05 |  | 5,034 | | 371,530 | 0.106613 | 0.122191 | 0.382932 |
| 38 | rs62075181 | T | C | 0.442435 | | 0.107706 | 3.99E-05 |  | 5,034 | | 371,530 | 0.037226 | 0.039033 | 0.340227 |
| 39 | rs6785758 | A | G | 0.364884 | | 0.081055 | 6.74E-06 |  | 5,034 | | 371,530 | 0.029843 | 0.028702 | 0.298464 |
| 40 | rs7242091 | G | A | -0.27487 | | 0.064947 | 2.31E-05 |  | 5,034 | | 371,530 | 0.015432 | 0.021546 | 0.473861 |
| 41 | rs72682840 | T | C | -0.62632 | | 0.151106 | 3.40E-05 |  | 5,034 | | 371,530 | -0.07557 | 0.040944 | 0.064937 |
| 42 | rs73237426 | T | A | 0.462271 | | 0.106278 | 1.36E-05 |  | 5,034 | | 371,530 | 0.003189 | 0.03918 | 0.935135 |
| 43 | rs73284905 | A | G | 0.30827 | | 0.070683 | 1.29E-05 |  | 5,034 | | 371,530 | -0.00276 | 0.024059 | 0.908596 |
| 44 | rs76196487 | G | A | 0.848037 | | 0.200174 | 2.27E-05 |  | 5,034 | | 371,530 | 0.025454 | 0.086912 | 0.769618 |
| 45 | rs7703397 | C | A | 0.311465 | | 0.070007 | 8.62E-06 |  | 5,034 | | 371,530 | -0.02708 | 0.021733 | 0.212727 |
| 46 | rs7823034 | A | G | -0.36298 | | 0.079897 | 5.54E-06 |  | 5,034 | | 371,530 | -0.03389 | 0.027902 | 0.22457 |
| 47 | rs79339719 | A | C | 0.606856 | | 0.144127 | 2.55E-05 |  | 5,034 | | 371,530 | 0.029679 | 0.054787 | 0.588013 |
| 48 | rs80101832 | G | A | -0.29745 | | 0.071367 | 3.07E-05 |  | 5,034 | | 371,530 | -0.02034 | 0.022047 | 0.356274 |
| 49 | rs8088539 | A | G | -0.39191 | | 0.084682 | 3.69E-06 |  | 5,034 | | 371,530 | 0.00644 | 0.029986 | 0.829946 |
| 50 | rs8096446 | C | T | -0.37934 | | 0.091908 | 3.67E-05 |  | 5,034 | | 371,530 | -0.01071 | 0.027412 | 0.696093 |
| 51 | rs9653729 | C | T | 0.282481 | | 0.06808 | 3.34E-05 |  | 5,034 | | 371,530 | 0.008732 | 0.022889 | 0.70282 |

SNP, single nucleotide polymorphism; EA, effect allele; OA, other allele; SE, standard error; AIT, autoimmune thyroiditis; UC, ulcerative colitis.

**Supplementary Table S2.3** SNPs from GWAS on AIT and CD.

|  |  | | | | **Exposure (AIT)** | | |  | | **Outcome (CD)** | | | | |
| --- | --- | --- | --- | --- | --- | --- | --- | --- | --- | --- | --- | --- | --- | --- |
|  | **SNP** | **EA** | **OA** | **β** | | **SE** | ***p* value** |  | **Case** | | **Control** | **β** | **SE** | ***p* value** |
| 1 | rs10484431 | G | A | 0.05043 | | 0.007175 | 2.08E-12 |  | 77,497 | | 299,780 | -0.00039 | 0.07951 | 0.996126 |
| 2 | rs11567705 | G | C | -0.04273 | | 0.006178 | 4.63E-12 |  | 77,497 | | 299,780 | -0.08021 | 0.068185 | 0.239425 |
| 3 | rs11618582 | G | A | -0.04501 | | 0.006135 | 2.17E-13 |  | 77,497 | | 299,780 | 0.002562 | 0.067526 | 0.969729 |
| 4 | rs11757605 | A | G | 0.056588 | | 0.00689 | 2.16E-16 |  | 77,497 | | 299,780 | 0.179449 | 0.077423 | 0.020461 |
| 5 | rs11838131 | G | A | -0.03199 | | 0.00584 | 4.33E-08 |  | 77,497 | | 299,780 | -0.02821 | 0.064602 | 0.66232 |
| 6 | rs1271066 | A | G | 0.040851 | | 0.006963 | 4.45E-09 |  | 77,497 | | 299,780 | 0.040411 | 0.076644 | 0.598015 |
| 7 | rs138481252 | T | C | -0.07058 | | 0.01198 | 3.82E-09 |  | 77,497 | | 299,780 | -0.05038 | 0.131517 | 0.701678 |
| 8 | rs1837253 | C | T | 0.038514 | | 0.006854 | 1.92E-08 |  | 77,497 | | 299,780 | -0.01631 | 0.0753 | 0.828568 |
| 9 | rs2197415 | G | T | 0.039285 | | 0.006294 | 4.32E-10 |  | 77,497 | | 299,780 | 0.013388 | 0.069424 | 0.847081 |
| 10 | rs2281917 | C | G | -0.04645 | | 0.008453 | 3.92E-08 |  | 77,497 | | 299,780 | -0.20767 | 0.092255 | 0.024384 |
| 11 | rs3757387 | C | T | 0.044001 | | 0.005855 | 5.71E-14 |  | 77,497 | | 299,780 | 0.090188 | 0.064769 | 0.163786 |
| 12 | rs3764 | C | T | -0.0439 | | 0.006712 | 6.16E-11 |  | 77,497 | | 299,780 | -0.0618 | 0.073985 | 0.403524 |
| 13 | rs429358 | C | T | -0.05146 | | 0.007614 | 1.40E-11 |  | 77,497 | | 299,780 | 0.015838 | 0.0837 | 0.849921 |
| 14 | rs62404122 | C | G | -0.10208 | | 0.010361 | 6.70E-23 |  | 77,497 | | 299,780 | -0.27039 | 0.112825 | 0.016549 |
| 15 | rs7590672 | C | T | -0.08179 | | 0.013332 | 8.54E-10 |  | 77,497 | | 299,780 | -0.02306 | 0.149196 | 0.877192 |
| 16 | rs7731626 | A | G | -0.03849 | | 0.006483 | 2.92E-09 |  | 77,497 | | 299,780 | -0.05198 | 0.071151 | 0.465088 |
| 17 | rs79918045 | A | G | 0.06783 | | 0.009409 | 5.62E-13 |  | 77,497 | | 299,780 | -0.03483 | 0.106297 | 0.743159 |
| 18 | rs846976 | C | A | 0.03818 | | 0.006568 | 6.14E-09 |  | 77,497 | | 299,780 | 0.041442 | 0.073473 | 0.572723 |
| 19 | rs9258945 | A | G | 0.067591 | | 0.005863 | 9.50E-31 |  | 77,497 | | 299,780 | 0.076133 | 0.064894 | 0.240721 |
| 20 | rs9549289 | G | A | -0.03986 | | 0.007191 | 2.98E-08 |  | 77,497 | | 299,780 | -0.06184 | 0.079511 | 0.436748 |

SNP, single nucleotide polymorphism; EA, effect allele; OA, other allele; SE, standard error; AIT, autoimmune thyroiditis; CD, Crohn's disease.

**Supplementary Table S2.4** SNPs from GWAS on IBD and AIT.

|  |  | | | | **Exposure (IBD)** | | |  | | **Outcome (AIT)** | | | | |
| --- | --- | --- | --- | --- | --- | --- | --- | --- | --- | --- | --- | --- | --- | --- |
|  | **SNP** | **EA** | **OA** | **β** | | **SE** | ***p* value** |  | **Case** | | **Control** | **β** | **SE** | ***p* value** |
| 1 | rs10737481 | G | T | 0.144709 | | 0.016387 | 1.04E-18 |  | 489 | | 320,703 | -0.0438555 | 0.0641278 | 0.494054 |
| 2 | rs10807943 | C | T | -0.34122 | | 0.030745 | 1.28E-28 |  | 489 | | 320,703 | 0.175972 | 0.131549 | 0.180996 |
| 3 | rs10931828 | T | C | -0.09523 | | 0.016588 | 9.43E-09 |  | 489 | | 320,703 | 0.0241405 | 0.0645147 | 0.708265 |
| 4 | rs117115824 | T | A | -0.16683 | | 0.029779 | 2.12E-08 |  | 489 | | 320,703 | -0.0119815 | 0.112702 | 0.915335 |
| 5 | rs11771806 | T | C | 0.135166 | | 0.024712 | 4.51E-08 |  | 489 | | 320,703 | -0.0244618 | 0.100534 | 0.807758 |
| 6 | rs12132298 | C | T | -0.16281 | | 0.020651 | 3.18E-15 |  | 489 | | 320,703 | -0.126969 | 0.0781486 | 0.104225 |
| 7 | rs12536069 | C | T | 0.252018 | | 0.030335 | 9.74E-17 |  | 489 | | 320,703 | -0.23367 | 0.129281 | 0.0706903 |
| 8 | rs12755372 | C | A | -0.11737 | | 0.019331 | 1.27E-09 |  | 489 | | 320,703 | -0.0318256 | 0.0745699 | 0.669533 |
| 9 | rs13165038 | C | T | -0.13114 | | 0.018048 | 3.69E-13 |  | 489 | | 320,703 | 0.00541813 | 0.0697717 | 0.938102 |
| 10 | rs1882597 | C | A | 0.203803 | | 0.036469 | 2.29E-08 |  | 489 | | 320,703 | 0.305562 | 0.154391 | 0.0477991 |
| 11 | rs191615076 | A | C | 0.246276 | | 0.039372 | 3.97E-10 |  | 489 | | 320,703 | -0.118685 | 0.169877 | 0.48477 |
| 12 | rs1986500 | A | G | 0.099924 | | 0.016541 | 1.53E-09 |  | 489 | | 320,703 | 0.00935782 | 0.0648878 | 0.88533 |
| 13 | rs2149560 | T | G | 0.12233 | | 0.016549 | 1.44E-13 |  | 489 | | 320,703 | 0.00230877 | 0.0646038 | 0.971492 |
| 14 | rs2836883 | A | G | -0.14097 | | 0.019459 | 4.34E-13 |  | 489 | | 320,703 | 0.0810317 | 0.0742986 | 0.275439 |
| 15 | rs3736162 | C | G | -0.10841 | | 0.018467 | 4.34E-09 |  | 489 | | 320,703 | -0.0788438 | 0.0713029 | 0.268831 |
| 16 | rs376365394 | T | C | 0.245757 | | 0.039053 | 3.11E-10 |  | 489 | | 320,703 | -0.166282 | 0.168404 | 0.323445 |
| 17 | rs3827023 | T | C | -0.14229 | | 0.021572 | 4.22E-11 |  | 489 | | 320,703 | 0.00200964 | 0.081832 | 0.980407 |
| 18 | rs4372078 | G | T | 0.121229 | | 0.018993 | 1.74E-10 |  | 489 | | 320,703 | -0.0631624 | 0.0730456 | 0.387204 |
| 19 | rs4676410 | A | G | 0.165787 | | 0.018097 | 5.15E-20 |  | 489 | | 320,703 | -0.0290026 | 0.0727427 | 0.690114 |
| 20 | rs4730275 | T | G | -0.11374 | | 0.017627 | 1.10E-10 |  | 489 | | 320,703 | -0.0639776 | 0.0680593 | 0.347203 |
| 21 | rs4807543 | T | G | 0.23941 | | 0.043471 | 3.64E-08 |  | 489 | | 320,703 | -0.287838 | 0.181019 | 0.111813 |
| 22 | rs56893428 | T | C | 0.091268 | | 0.016465 | 2.97E-08 |  | 489 | | 320,703 | 0.0111366 | 0.0647209 | 0.863382 |
| 23 | rs6017342 | C | A | 0.122771 | | 0.016616 | 1.48E-13 |  | 489 | | 320,703 | 0.0987399 | 0.0646732 | 0.126823 |
| 24 | rs67927699 | C | G | 0.102968 | | 0.016812 | 9.09E-10 |  | 489 | | 320,703 | 0.134144 | 0.0664219 | 0.043428 |
| 25 | rs895123 | G | C | 0.142454 | | 0.020129 | 1.47E-12 |  | 489 | | 320,703 | 0.0624499 | 0.0811707 | 0.441676 |
| 26 | rs9607629 | G | A | -0.17825 | | 0.026026 | 7.43E-12 |  | 489 | | 320,703 | -0.134696 | 0.0967613 | 0.16391 |
| 27 | rs9617090 | T | C | -0.12894 | | 0.017137 | 5.30E-14 |  | 489 | | 320,703 | -0.0506828 | 0.0664048 | 0.445321 |
| 28 | rs9988642 | C | T | -0.45969 | | 0.044693 | 8.19E-25 |  | 489 | | 320,703 | -0.132774 | 0.155963 | 0.394593 |

SNP, single nucleotide polymorphism; EA, effect allele; OA, other allele; SE, standard error; IBD, inflammatory bowel disease; AIT, autoimmune thyroiditis.

**Supplementary Table S2.5** SNPs from GWAS on UC and AIT.

|  |  | | | | **Exposure (UC)** | | |  | | **Outcome (AIT)** | | | | |
| --- | --- | --- | --- | --- | --- | --- | --- | --- | --- | --- | --- | --- | --- | --- |
|  | **SNP** | **EA** | **OA** | **β** | | **SE** | ***p* value** |  | **Case** | | **Control** | **β** | **SE** | ***p* value** |
| 1 | rs10737481 | G | T | 0.177217 | | 0.020053 | 9.78E-19 |  | 489 | | 320,703 | -0.0438555 | 0.0641278 | 0.494054 |
| 2 | rs10799837 | A | G | -0.11875 | | 0.020212 | 4.22E-09 |  | 489 | | 320,703 | 0.0684085 | 0.0646773 | 0.290196 |
| 3 | rs10807943 | C | T | -0.31354 | | 0.037902 | 1.31E-16 |  | 489 | | 320,703 | 0.175972 | 0.131549 | 0.180996 |
| 4 | rs11209026 | A | G | -0.38229 | | 0.053654 | 1.04E-12 |  | 489 | | 320,703 | -0.133725 | 0.155737 | 0.390528 |
| 5 | rs12536069 | C | T | 0.245799 | | 0.037218 | 4.00E-11 |  | 489 | | 320,703 | -0.23367 | 0.129281 | 0.0706903 |
| 6 | rs12736494 | A | G | -0.13767 | | 0.023245 | 3.17E-09 |  | 489 | | 320,703 | -0.0187613 | 0.0730546 | 0.797324 |
| 7 | rs13024106 | A | G | -0.1159 | | 0.020206 | 9.69E-09 |  | 489 | | 320,703 | 0.00403513 | 0.0650734 | 0.950556 |
| 8 | rs13165038 | C | T | -0.13335 | | 0.022105 | 1.61E-09 |  | 489 | | 320,703 | 0.00541813 | 0.0697717 | 0.938102 |
| 9 | rs1878668 | G | T | 0.119883 | | 0.0206 | 5.90E-09 |  | 489 | | 320,703 | -0.058792 | 0.0651469 | 0.366817 |
| 10 | rs34236350 | T | C | 0.17698 | | 0.022135 | 1.29E-15 |  | 489 | | 320,703 | -0.043522 | 0.07288 | 0.550392 |
| 11 | rs4263839 | G | A | 0.12357 | | 0.022366 | 3.30E-08 |  | 489 | | 320,703 | -0.0566636 | 0.070316 | 0.420334 |
| 12 | rs4817986 | T | G | -0.15786 | | 0.023884 | 3.86E-11 |  | 489 | | 320,703 | 0.0814832 | 0.0742746 | 0.272618 |
| 13 | rs56086041 | G | T | -0.17927 | | 0.029056 | 6.84E-10 |  | 489 | | 320,703 | 0.0289542 | 0.089637 | 0.746683 |
| 14 | rs6017342 | C | A | 0.149349 | | 0.020348 | 2.14E-13 |  | 489 | | 320,703 | 0.0987399 | 0.0646732 | 0.126823 |
| 15 | rs6089926 | T | C | -0.16264 | | 0.025723 | 2.57E-10 |  | 489 | | 320,703 | 0.0523218 | 0.0793728 | 0.509773 |
| 16 | rs6967335 | A | C | -0.15636 | | 0.020383 | 1.70E-14 |  | 489 | | 320,703 | -0.0551239 | 0.0647672 | 0.394709 |
| 17 | rs7865719 | G | A | 0.123757 | | 0.020261 | 1.01E-09 |  | 489 | | 320,703 | 0.00262858 | 0.064577 | 0.967531 |
| 18 | rs7930763 | A | G | 0.133479 | | 0.020125 | 3.30E-11 |  | 489 | | 320,703 | 0.0916204 | 0.0647035 | 0.156775 |
| 19 | rs9607629 | G | A | -0.20304 | | 0.032047 | 2.36E-10 |  | 489 | | 320,703 | -0.134696 | 0.0967613 | 0.16391 |
| 20 | rs9617090 | T | C | -0.16385 | | 0.021029 | 6.60E-15 |  | 489 | | 320,703 | -0.0506828 | 0.0664048 | 0.445321 |

SNP, single nucleotide polymorphism; EA, effect allele; OA, other allele; SE, standard error; UC, ulcerative colitis; AIT, autoimmune thyroiditis.

**Supplementary Table S2.6** SNPs from GWAS on CD and AIT.

|  |  | | | | **Exposure (CD)** | | |  | | **Outcome (AIT)** | | | | |
| --- | --- | --- | --- | --- | --- | --- | --- | --- | --- | --- | --- | --- | --- | --- |
|  | **SNP** | **EA** | **OA** | **β** | | **SE** | ***p* value** |  | **Case** | | **Control** | **β** | **SE** | ***p* value** |
| 1 | rs10484431 | G | A | 0.0504299 | | 0.00717451 | 2.08E-12 |  | 489 | | 320,703 | -0.000386059 | 0.0795102 | 0.996126 |
| 2 | rs11567705 | G | C | -0.042728 | | 0.00617769 | 4.63E-12 |  | 489 | | 320,703 | -0.0802143 | 0.0681847 | 0.239425 |
| 3 | rs11618582 | G | A | -0.0450149 | | 0.00613457 | 2.17E-13 |  | 489 | | 320,703 | 0.00256247 | 0.0675262 | 0.969729 |
| 4 | rs11757605 | A | G | 0.0565883 | | 0.00689042 | 2.16E-16 |  | 489 | | 320,703 | 0.179449 | 0.0774225 | 0.0204607 |
| 5 | rs11838131 | G | A | -0.0319867 | | 0.00584041 | 4.33E-08 |  | 489 | | 320,703 | -0.0282125 | 0.064602 | 0.66232 |
| 6 | rs1271066 | A | G | 0.0408509 | | 0.00696316 | 4.45E-09 |  | 489 | | 320,703 | 0.040411 | 0.0766438 | 0.598015 |
| 7 | rs138481252 | T | C | -0.0705816 | | 0.0119799 | 3.82E-09 |  | 489 | | 320,703 | -0.0503785 | 0.131517 | 0.701678 |
| 8 | rs1837253 | C | T | 0.038514 | | 0.00685379 | 1.92E-08 |  | 489 | | 320,703 | -0.0163053 | 0.0752997 | 0.828568 |
| 9 | rs2197415 | G | T | 0.0392852 | | 0.0062938 | 4.32E-10 |  | 489 | | 320,703 | 0.013388 | 0.069424 | 0.847081 |
| 10 | rs2281917 | C | G | -0.0464463 | | 0.00845334 | 3.92E-08 |  | 489 | | 320,703 | -0.20767 | 0.0922554 | 0.0243837 |
| 11 | rs3757387 | C | T | 0.0440012 | | 0.00585542 | 5.71E-14 |  | 489 | | 320,703 | 0.0901879 | 0.0647692 | 0.163786 |
| 12 | rs3764 | C | T | -0.0438966 | | 0.00671212 | 6.16E-11 |  | 489 | | 320,703 | -0.0618033 | 0.0739854 | 0.403524 |
| 13 | rs429358 | C | T | -0.0514607 | | 0.00761445 | 1.40E-11 |  | 489 | | 320,703 | 0.0158376 | 0.0836998 | 0.849921 |
| 14 | rs62404122 | C | G | -0.102076 | | 0.0103606 | 6.70E-23 |  | 489 | | 320,703 | -0.270392 | 0.112825 | 0.0165493 |
| 15 | rs7590672 | C | T | -0.0817874 | | 0.0133321 | 8.54E-10 |  | 489 | | 320,703 | -0.0230552 | 0.149196 | 0.877192 |
| 16 | rs7731626 | A | G | -0.038485 | | 0.00648335 | 2.92E-09 |  | 489 | | 320,703 | -0.0519752 | 0.0711507 | 0.465088 |
| 17 | rs79918045 | A | G | 0.0678301 | | 0.00940868 | 5.62E-13 |  | 489 | | 320,703 | -0.0348304 | 0.106297 | 0.743159 |
| 18 | rs846976 | C | A | 0.0381798 | | 0.00656826 | 6.14E-09 |  | 489 | | 320,703 | 0.041442 | 0.0734729 | 0.572723 |
| 19 | rs9258945 | A | G | 0.0675909 | | 0.00586306 | 9.50E-31 |  | 489 | | 320,703 | 0.076133 | 0.0648942 | 0.240721 |
| 20 | rs9549289 | G | A | -0.0398566 | | 0.00719077 | 2.98E-08 |  | 489 | | 320,703 | -0.0618354 | 0.079511 | 0.436748 |

SNP, single nucleotide polymorphism; EA, effect allele; OA, other allele; SE, standard error; CD, Crohn's disease; AIT, autoimmune thyroiditis.

**Supplementary Table S3** The results of MR-Egger intercept analysis.

| **Exposure** | **Outcome** | **Egger_intercept** | **SE** | ***p* value** |
| --- | --- | --- | --- | --- |
| AIT | IBD | 0.002172348 | 0.010229335 | 0.832721911 |
| AIT | UC | 0.010643113 | 0.011882643 | 0.374799837 |
| AIT | CD | -0.008857224 | 0.021051465 | 0.67571161 |
| IBD | AIT | 0.068993667 | 0.045154538 | 0.13860161 |
| UC | AIT | 0.010680722 | 0.063854825 | 0.8690258 |
| CD | AIT | -0.037521663 | 0.063175035 | 0.55994872 |

AIT, autoimmune thyroiditis; IBD, inflammatory bowel disease; UC, ulcerative colitis; CD, Crohn's disease.

**Supplementary Table S4** The results of heterogeneity analysis.

| **Exposure** | **Outcome** | **Method** | **Q** | **Q_df** | **Q_*p* val** |
| --- | --- | --- | --- | --- | --- |
| AIT | IBD | MR Egger | 50.35341739 | 48 | 0.38048267 |
| AIT | IBD | Inverse variance weighted | 50.40072725 | 49 | 0.417841445 |
| AIT | UC | MR Egger | 47.91319479 | 49 | 0.517178362 |
| AIT | UC | Inverse variance weighted | 48.71544755 | 50 | 0.524996988 |
| AIT | CD | MR Egger | 47.33192832 | 51 | 0.620125815 |
| AIT | CD | Inverse variance weighted | 47.50895169 | 52 | 0.65092851 |
| IBD | AIT | MR Egger | 28.06636696 | 26 | 0.355200314 |
| IBD | AIT | Inverse variance weighted | 30.58652917 | 27 | 0.288453381 |
| UC | AIT | MR Egger | 18.43094374 | 18 | 0.427621163 |
| UC | AIT | Inverse variance weighted | 18.45959137 | 19 | 0.491961011 |
| CD | AIT | MR Egger | 11.34939063 | 18 | 0.878946292 |
| CD | AIT | Inverse variance weighted | 11.70214563 | 19 | 0.897959136 |

AIT, autoimmune thyroiditis; IBD, inflammatory bowel disease; UC, ulcerative colitis; CD, Crohn's disease.
